# The SCARECROW‐LIKE transcription factor from *Populus davidiana* × *P. bolleana* simultaneously improved drought tolerance and plant growth through acetylation‐dependent mechanisms

**DOI:** 10.1111/pbi.70185

**Published:** 2025-06-09

**Authors:** Pengyu Wang, Xue Yang, Shilin Sun, Jingxin Wang, Jingwen Wang, Xiaofu Li, Dandan Li, Yucheng Wang

**Affiliations:** ^1^ College of Forestry Shenyang Agricultural University Shenyang China; ^2^ Key Laboratory of Forest Tree Genetics Breeding and Cultivation of Liaoning Province Shenyang China

**Keywords:** poplar, GRAS family, PdbSCL1, drought stress, acetylation, protein interaction

## Abstract

The GAI‐RGA‐and‐SCR (GRAS) family of plant‐specific transcription factors (TFs) is essential for development and stress tolerance, but their role in drought resistance and growth remains unclear. Here, we characterized the SCARECROW‐LIKE (SCL) TF *PdbSCL1* from *Populus davidiana × P. bolleana*, examining its role in drought response and growth. Overexpression of *PdbSCL1* improved drought tolerance and growth, while knockout lines exhibited decreased drought tolerance and growth. Under normal conditions, overexpression lines showed a 16.7% increase in height and 14.6% in fresh weight compared with wild‐type (WT). Under drought conditions, these increases reached 32.2% and 79.5%, respectively. PdbSCL1 regulates gene expression by binding to DNA motifs such as ABRE (‘CACGTG’), PCF (‘TGGGCC’), and NFY (‘CCAAT’). Drought‐induced acetylation of PdbSCL1 at key lysine residues (106 and 444) is critical for its regulatory function. Mutations at these sites impair its ability to regulate gene expression, leading to reduced drought tolerance and growth. PdbSCL1 also interacts with a histone acetyltransferase 3 (PdbHAG3), which catalyses its acetylation, further enhancing drought resilience and growth. These findings highlight the essential role of PdbSCL1 acetylation in both drought response and growth promotion, suggesting its potential application in molecular breeding to improve drought tolerance and growth in poplar.

## Introduction

The GRAS gene family is a plant‐specific transcription factor (TF) family named after its three initially identified members: gibberellin acid insensitive (GAI), repressor of GA1 (RGA), and scarecrow (SCR), which are typically 360–850 amino acids in length (Yang *et al*., 2021). GRAS proteins have a variable N‐terminal region, which includes homopolymeric stretches of specific amino acids, intrinsically disordered regions, and a conserved DELLA domain. They also possess a highly conserved C‐terminal region known as the GRAS domain (Jaiswal *et al*., 2022; Waseem *et al*., 2022). The GRAS domain consists of five distinct sequence motifs: leucine heptad repeat I (LHRI), leucine heptad repeat II (LHRII), VHIID, PFYRE, and SAW (He *et al*., 2022). The structure of the GRAS domain is characterized by α‐helices from the leucine‐rich region I (LRI) subdomain forming the cap, with two additional helices from the PFYRE subdomain. The remaining subdomains form the core of the structure. The region between the LRI cap and the VHIID core is missing, suggesting potential movement in this area (Hofmann, 2016). These motifs are critical for facilitating protein–protein interactions and play a significant role in the functional diversity of GRAS TFs across various physiological processes in plants. The function of GRAS family proteins is found to be involved in regulating gibberellin signalling (Torres‐Galea *et al*., 2013), root and shoot development (Heo *et al*., 2011), starch biosynthesis (Cai *et al*., 2017), male gametogenesis (Morohashi *et al*., 2003), asymmetric cell division, cell proliferation, and plant growth (Bisht *et al*., 2023; Dutta *et al*., 2021; Hirano *et al*., 2017; Yu *et al*., 2023). Furthermore, GRAS also plays an important role in abiotic stress tolerance, such as salinity, drought, and osmotic stress (Jaiswal *et al*., 2022).

*SCARECROW*‐*LIKE* (*SCL*) TF, a member of the GRAS gene family, encodes that are also involved in plant development and abiotic stress (Yang *et al*., 2021). However, there is still very limited information about the DNA motifs bound by the SCL TF and its target genes, which are crucial for a deeper understanding of the SCL TF's function.

Post‐translational modifications (PTMs), catalysed by various enzymes, serve as a regulatory mechanism for modulating protein activity, localization, expression, and interactions with other cellular molecules, significantly increasing protein diversity (Li *et al*., 2023; Zhu *et al*., 2022). Among PTMs in eukaryotes, protein acetylation and phosphorylation stand out as the most prevalent forms that influence protein functions (Uhrig *et al*., 2019). Protein acetylation encompasses both histone and non‐histone proteins. Specifically, histone acetylation is closely associated with gene transcription, likely due to its role in relaxing chromatin structure and facilitating access for transcription machinery. Additionally, acetylation of certain lysine residues may serve as recognition sites for TFs (Narita *et al*., 2019). Non‐histone targets of lysine acetylation predominantly include TFs and essential cellular proteins, such as heat shock proteins and cortactin (Zhang *et al*., 2007). Consequently, lysine acetylation is crucial not only for transcriptional regulation but also for processes related to signal transduction and cellular transport. Furthermore, it serves as a key metabolic regulatory signal, especially at the mitochondrial level (Narita *et al*., 2019; Shvedunova and Akhtar, 2022).

Acetylation is a key epigenetic modification that regulates TFs, affecting plant resistance to abiotic or biotic stresses. The acetylation and deacetylation of genes and TFs are vital for plants' responses to abiotic stresses like drought and salinity, as well as biotic stresses such as pathogen infections. For example, Histone Deacetylase 9 (HDA9) plays a key role in stress resistance by removing acetylation modifications from the TF WRKY53, thereby inhibiting its transcriptional activity. Conversely, WRKY53 can negatively regulate HDA9 activity, creating an antagonistic relationship between the two proteins in response to stress (Zheng *et al*., 2020). Acetylation also enhances plant tolerance to osmotic stress by up‐regulating the expression of the *AhDREB1* gene (Zhang *et al*., 2018). In maize, acetylation of histones H3 and H4 promotes the rapid induction of *ZmDREB2A* expression in response to osmotic stress (Zhao *et al*., 2014). Furthermore, under salt stress, histone acetylation significantly influences the transcriptional activation of the *POX* gene, suggesting a close link between acetylation‐mediated gene expression and salt tolerance (Yolcu *et al*., 2016). Additionally, some studies on acetylation of TFs have been studied. For instance, in birch, the acetyltransferase BpPDCE23 acetylates a lysine residue on BpTCP20, enhancing its binding to the promoters of target genes, thereby improving salt tolerance (Liu *et al*., 2024). In *Rhododendron chrysanthum*, UV‐B stress resulted in the up‐regulation of acetylation at the K68 locus of the *RcTRP5* TF while simultaneously causing a dow‐nregulation of *RcTRP5* expression. These changes in RcTRP5 indirectly promoted the acetylation modifications of the cinnamyl alcohol dehydrogenase (CAD) and phenylalanine ammonia lyase (PAL) enzymes. Consequently, this led to an increase in the expression of the G‐type lignin site of the CAD enzyme and a down‐regulation of the K391 site of the PAL enzyme, ultimately resulting in elevated G‐type lignin expression and an increased G/S ratio (Gong *et al*., 2024). Despite previous research, it remains uncertain whether the SCL TF undergoes acetylation modifications. If such modifications do take place, their role in influencing the function of the SCL TF is still not fully understood.

In this study, we characterized a drought‐responsive SCL TF from *Populus davidiana* × *P*. *bolleana* (Shanxin poplar), designated PdbSCL1. Our findings indicate that PdbSCL1 not only accelerates growth but also confers significant drought tolerance. Furthermore, we investigated whether PdbSCL1 responds to drought stress through PTMs, focusing on phosphorylation, acetylation, and ubiquitination. We found that PdbSCL1 undergoes significant acetylation under drought stress, while the other modifications were less prominent. Additional investigations revealed that acetylation plays a crucial role in activating the target genes of PdbSCL1, making it essential for enhancing drought tolerance and promoting growth. This research provides novel insights into the mechanisms underlying drought tolerance in Shanxin poplar.

## Results

### Characterization of the sequence and expression profile of *PdbSCL1* in response to drought stress

Previously, we constructed a regulatory network of Shanxin poplar in response to drought and identified *PdbSCL1* as one of the upstream regulators of this network (Wang *et al*., 2022b). This suggests that it plays an important role in drought tolerance, leading us to select it for further study. To investigate the response of *PdbSCL1* to drought stress, qRT‐PCR was used to analyse the expression of *PdbSCL1* in the leaves of Shanxin poplar under PEG‐induced drought stress. The expression of *PdbSCL1* increased steadily, peaking after 9 h of stress before gradually declining. This suggests that *PdbSCL1* plays a role in the plant's response to drought stress (Figure S1A). Multiple sequence analysis revealed that PdbSCL1 contains several conserved regions characteristic of other GRAS family members, including the LEUCINE HEPTAD, LEUCINE HEPTAD H, PFYRE, and SAW motifs, indicating that these regions are important for the function of GRAS proteins (Figure S1B). Phylogenetic analysis indicated that PdbSCL1 is most closely related to AtSCL3 and PtGRAS1, suggesting that they may share similar biological functions (Figure S1C).

### PdbSCL1 enhances both drought tolerance and growth in transgenic plants

To investigate the function of *PdbSCL1*, we generated transgenic poplar lines that overexpress *PdbSCL1* and knockout lines lacking *PdbSCL1*. Six overexpression transgenic lines were successfully produced, from which three lines with relatively high expression levels, OE2, OE4, and OE5 (In the following studies, they were named OE1, OE2, OE3), were selected for further analysis based on qRT‐PCR and Western blotting results (Figure S2A–C). Using the CRISPR/Cas9 method, we also generated three PdbSCL1 knockout lines (*scl*), designated as *scl1*, *scl2*, and *scl3*, for further study. In *scl1* and *scl2*, a nucleotide insertion occurred in the CDS, while *scl3* exhibited a base deletion in the CDS. These insertions and deletions resulted in frame shift mutations in the protein‐CDSs, leading to the alteration of PdbSCL1 (Figure S2D).

**Figure 1:**
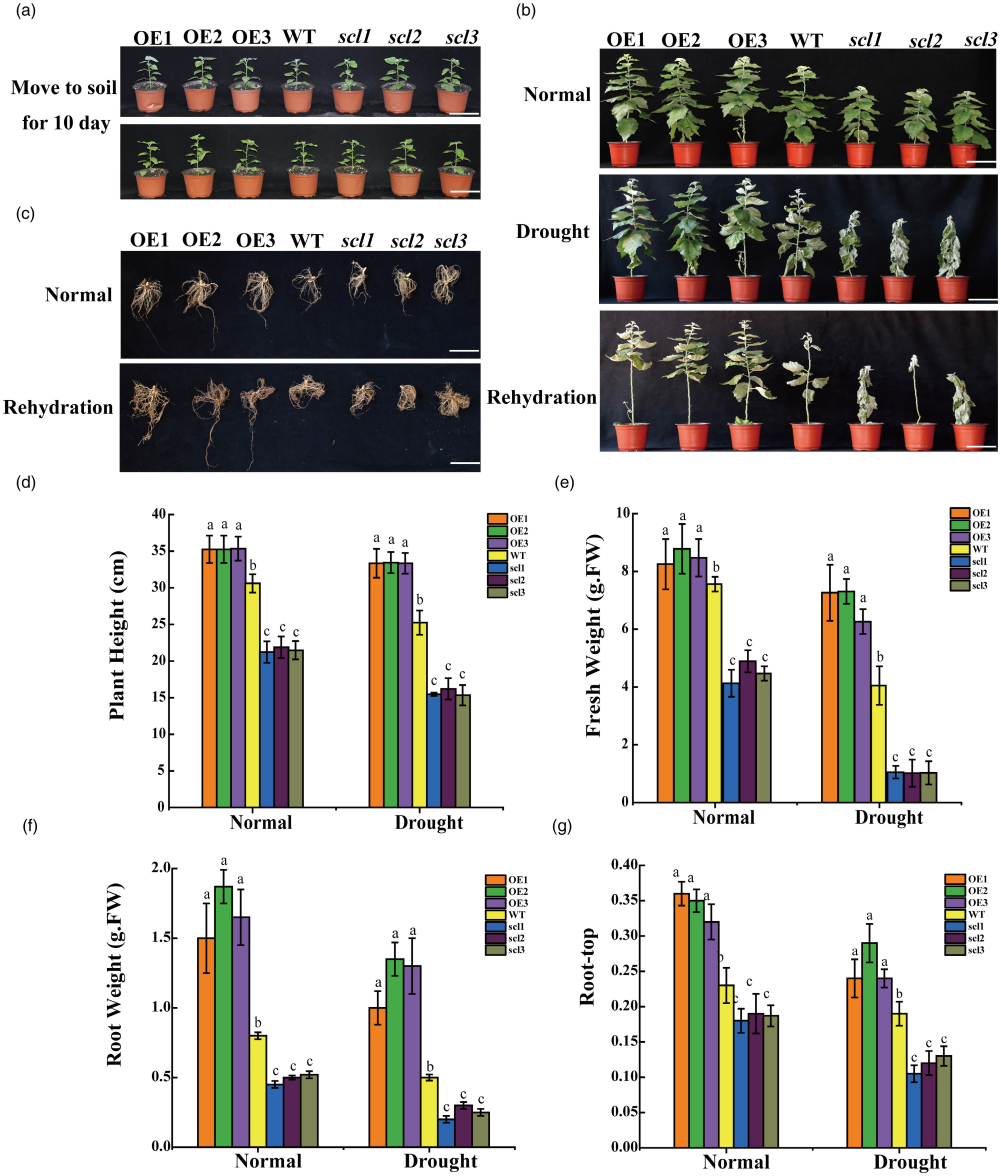
Determination of drought tolerance conferred by PdbSCL1. (a) Growth phenotypes of OE, WT, and *scl* lines under control and drought stress conditions. Scale bar = 5 cm. Wild‐type (WT) and transgenic poplar plantlets of similar sizes were used for the study. (b) After 30 days of normal growth, plants were not watered for 10 days and then rehydrated for 10 days; well‐watered plants served as controls. (c) Root phenotypes of OE, WT, and *scl* lines under control and drought stress conditions. Scale bar = 5 cm. (d) Analysis of plant height. (e) Analysis of total plant fresh weight. (f) Analysis of root weight. (g) Determination of root‐to‐shoot ratio. Key: OE: Shanxin poplar lines overexpressing *PdbSCL1*; *scl*: Shanxin poplar with *PdbSCL1* knockout induced by CRISPR/Cas9; WT, wild‐type plants. ‘Normal’ indicates plants grown under standard conditions; ‘drought’ indicates plants treated with no water for 10 days; ‘rehydration’ indicates plants watered well for 10 days after drought. Error bars represent standard deviation (SD) from three biological replicates, each containing 5–10 plantlets.

Plants of similar size, including those overexpressing and knocking out PbdSCL1 as well as WT plants, were transplanted into pots with soil for growth (Figure 1a). For the drought stress experiment, the plants underwent 10 days without watering, followed by 10 days of rehydration. They were assessed after both the drought stress and the rehydration periods, while the plants grown under normal conditions served as controls. Under normal growth conditions for 30 days, the OE lines exhibited the highest plant height, total fresh weight, root weight, and root‐to‐shoot ratio, followed by the WT and then the *scl* lines (Figure 1b–g). Compared with WT, the OE lines showed a 16.7% increase in plant height and a 14.6% increase in fresh weight (Figure 1a,d,e), indicating that PdbSCL1 plays a key role in promoting plant growth.

Furthermore, after experiencing drought stress for 10 days, the OE lines showed greater drought tolerance compared with WT, while the knockout lines were the least tolerant (Figure 1b). After rehydration, the OE lines resumed normal growth, whereas the WT and mutant lines failed to recover and died. The OE lines again displayed the highest total fresh weight, root weight, root‐to‐shoot ratio, followed by the WT and then the *PdbSCL1* knockout lines (Figure 1b–g). These results suggest that PdbSCL1 not only facilitates plant growth but also confers drought tolerance.

### PdbSCL1 facilitates physiological changes that enhance drought tolerance in plants

In this study, plants subjected to a drought treatment were deprived of water for 10 days, while well‐watered plants served as controls. Under normal growth conditions, all plants exhibited similar levels of MDA, reactive oxygen species (ROS), and electrolyte leakage (Figure S3). However, after drought treatment, all OE lines demonstrated significantly lower levels of MDA, ROS, and electrolyte leakage compared with WT plants. In contrast, the PdbSCL1 knockout lines (*scl1*, *scl2*, and *scl3*) also showed increased MDA, ROS, and electrolyte leakage compared with WT plants (Figure S3A–C).

Given that ROS levels were reduced in the OE lines under salt stress conditions, we further investigated the activity of ROS scavenging enzymes, specifically POD and SOD. Under normal conditions, there were no significant differences in SOD and POD activities among the various poplar lines (Figure S3D,E). Following drought treatment, the PdbSCL1‐OE lines exhibited significantly higher activities of SOD and POD compared with WT plants (Figure S3D,E), which also outperformed the mutant lines (*scl1*, *scl2*, and *scl3*). These results suggest that PdbSCL1 may mediate the activities of SOD and POD, thereby reducing ROS accumulation.

Additionally, proline levels were measured, revealing that the OE transgenic lines had the highest proline content, followed by WT plants, while the mutant lines had the lowest proline levels under drought stress (Figure S3F). These findings indicate that overexpression of PdbSCL1 leads to an accumulation of proline, contributing to improved drought tolerance.

### Integrative analysis of RNA‐seq and ChIP‐seq

To elucidate the molecular mechanism by which PdbSCL1 regulates drought tolerance, we conducted a transcriptomic analysis of *PdbSCL1* overexpression (OE) and WT plants after 10 days of drought stress (row data stored in NCBI with submission number NCBI GEO: PRJNA1134423). A total of 1632 differentially expressed genes were identified, with 157 genes up‐regulated and 1475 down‐regulated in response to PdPLC1 (Figure S4; Table S2).

**Figure 2:**
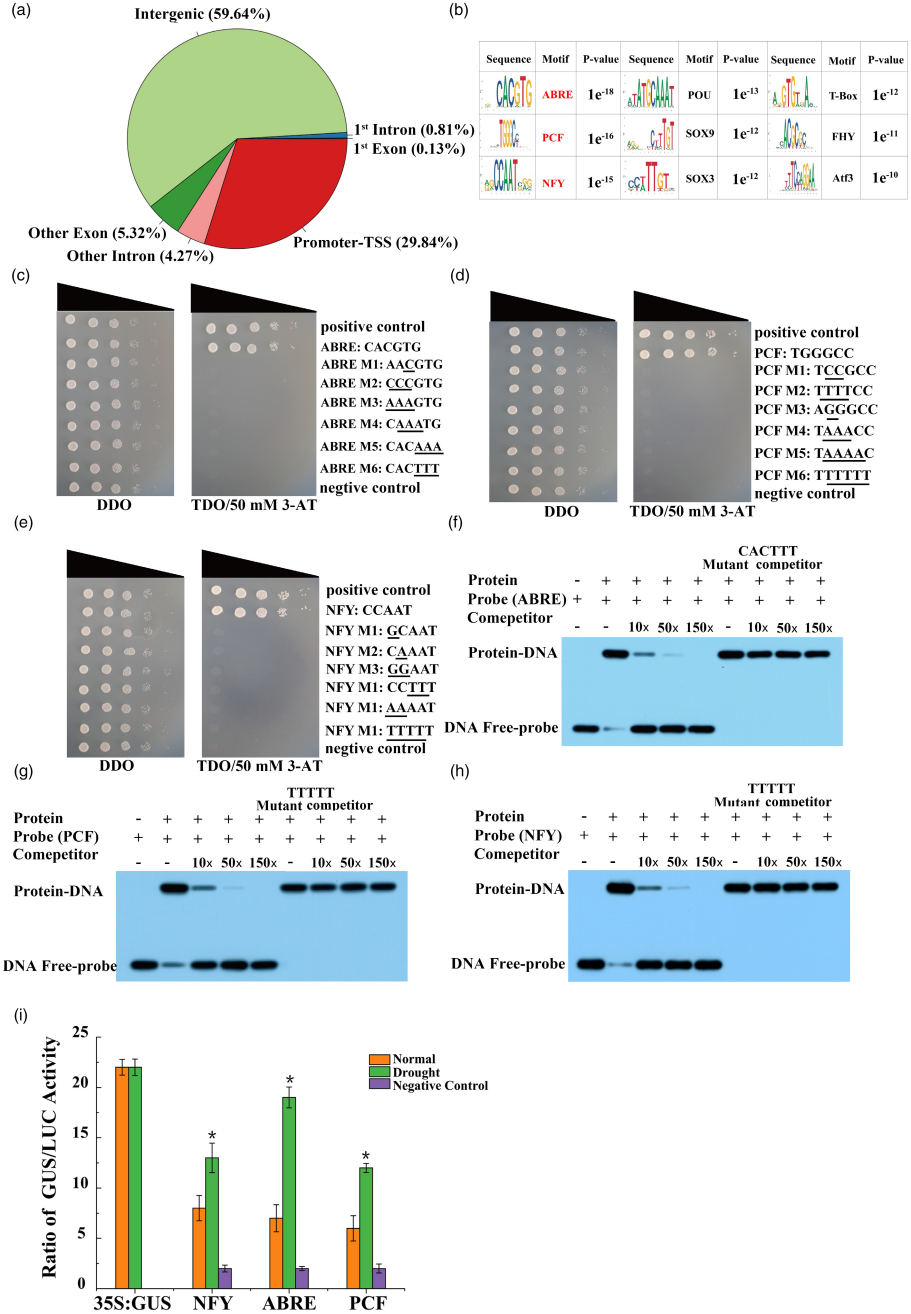
Identification of DNA motifs bound by PdbSCL1. (a) The distribution of peaks on different chromatin regions. (b) Conserved sequences potentially bound by PdbSCL1 were identified via ChIP‐seq. The motifs ABRE (‘CACGTG’), PCF (‘TGGGCC’), and NFY (‘CCAAT’) were selected for further binding verification. (c–e) The binding of PdbSCL1 to ABRE (c), PCF (d), and NFY (e) was confirmed using a Y1H assay. Transformed yeast dilutions (1^×^, 10^−1×^, 10^−2×^, 10^−3×^, 10^−4×^) were plated on synthetic dextrose (SD) media lacking leucine and tryptophan (DDO) and on SD media lacking leucine, tryptophan, and histidine (TDO) with 3‐AT. Positive control: p53HIS2 + pGAD‐Rec2‐53; Negative control: p53HIS2 + pGAD‐PdbSCL1. Mutated nucleotides are indicated by lines. (f–h) EMSA confirmed the binding of PdbSCL1 to ABRE (f), PCF (g), and NFY (h). Mutant competitors: the ABRE, PCR, and NFY were respectively mutated to sequences of ‘CACTT’, ‘TTTTT’ and ‘TTTTT’, and the sequences without labels were used as competitor. (i) The transcriptional activity of PdbSCL1 on the DNA motifs ABRE, PCF, and NFY. 35S:PdbSCL1 served as the effector. Each motif was fused with the 35S minimal promoter to drive GUS expression as reporters. Additionally, 35S:Luc was transiently transformed into Shanxin poplar with the effector and reporter to normalize transformation efficiency. The GUS to Luc ratio was measured to evaluate transcriptional activation. ‘Normal’ indicates standard conditions, while ‘Drought’ refers to plants treated with 20% PEG6000 for 9 h. The negative control included only the reporter. Asterisk (*) indicates statistical significance (*P* < 0.05).

To identify the binding regions and the genes directly regulated by PdbSCL1, we performed ChIP‐seq analysis (row data stored in NCBI with submission number: PRJNA1131963). The distribution of peaks revealed that 29.84% of the peaks were located within 3 kb upstream of the translation start site (Figure 2a). Given the absence of information regarding the transcription initiation site (TIS), we used the transcription start site (TSS) to define the promoter boundaries (Figure 2a). We predicted the motifs bound by PdSCL1 and identified nine conserved motifs (*P*‐value<e^−10^) (Figure 2b). To verify the binding capability of PdSCL1 to these motifs, we selected three conserved motifs with the smallest *P*‐values, including ABRE (‘CACGTG’), PCF (‘TGGGCC’), and NFY (‘CCAAT’) for further analysis.

Results from the yeast one‐hybrid (Y1H) assay demonstrated that PdSCL1 can bind to the ABRE, PCF, and NFY motifs, but not to their mutated forms (Figure 2c–e). To further confirm the binding of PdbSCL1 to these motifs, we conducted an EMSA. The results indicated that PdbSCL1 binds to these motifs, as evidenced by the presence of shifted bands, which decreased in intensity as competitor probes (unlabeled probes) were gradually added. In contrast, the mutated probes did not show binding with PdbSCL1 (Figure 2f–h).

Additionally, we assessed the transcriptional activation of the ABRE, PCF, and NFY motifs. PdbSCL1 driven by the CaMV 35S promoter served as the effector, while each motif drove the expression of a GUS reporter gene. Both the effector and reporter constructs were transiently transformed into Shanxin poplar, along with a luciferase construct driven by the CaMV 35S promoter (35S: Luc). The GUS/Luc ratio was subsequently measured. The results confirmed that PdbSCL1 can bind to the ABRE, PCF, and NFY motifs, leading to the activation of gene expression (Figure 2i).

### Drought stress tolerance genes potentially regulated by PdbSCL1 identified through RNA‐seq and ChIP‐seq analyses

**Figure 3:**
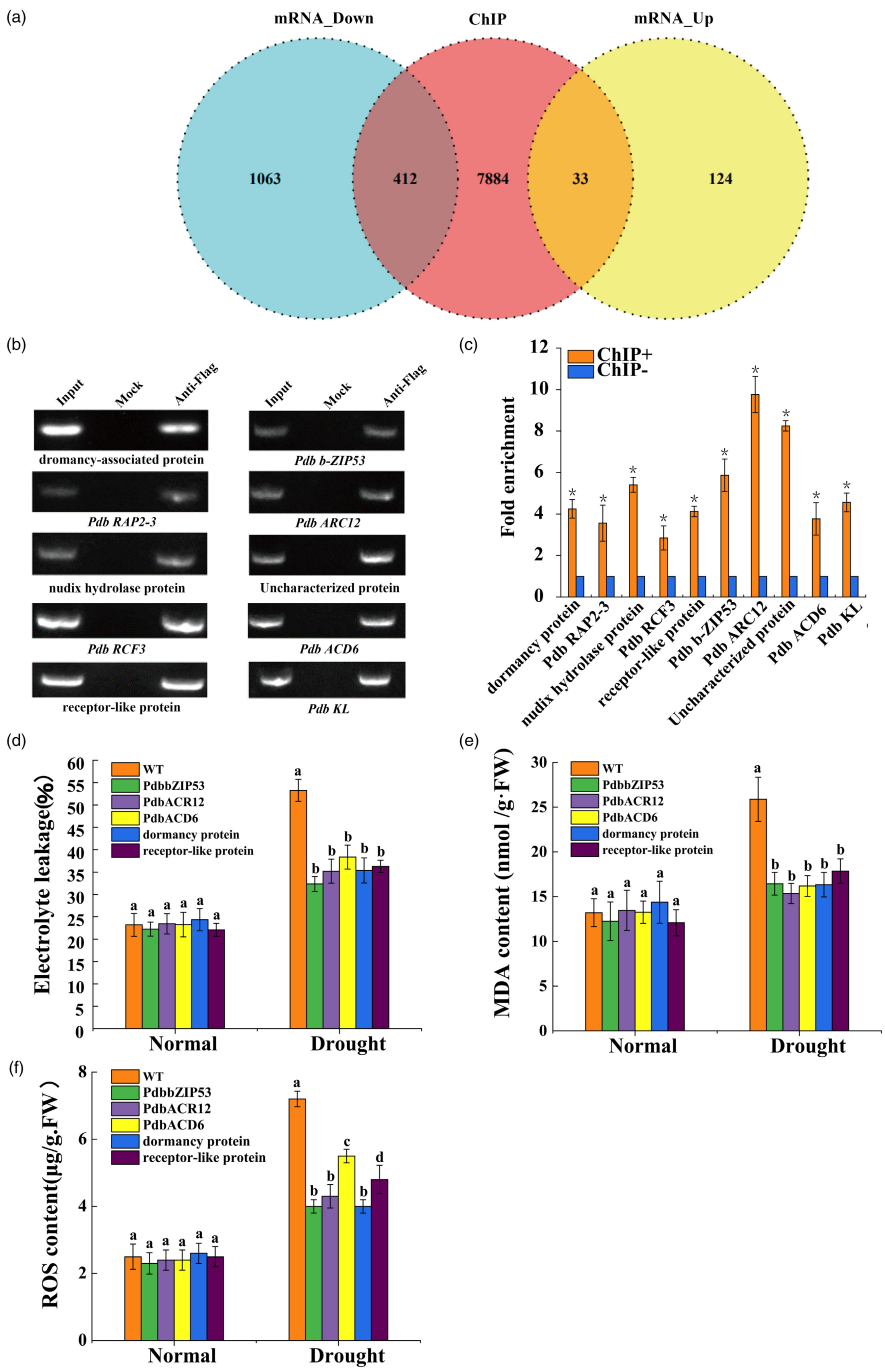
Joint analysis of ChIP‐seq and RNA‐seq. (a) Venn diagram showing the distribution of differentially expressed genes from the combined RNA‐seq and ChIP‐seq analysis. (b, c) Downstream target genes regulated by PdbSCL1 were analysed using ChIP–PCR and ChIP–qPCR. The truncated promoters harbouring ABRE, PC, and NFY were used for PCR. ‘Input’ served as the positive control, while ‘Mock’ indicates the negative control without antibodies. ChIP+: Anti‐Flag denotes the ChIP product immunoprecipitated with Flag antibody. ChIP−: Water was used as a control in place of the antibody for immunoprecipitating chromatin. The relative fold enrichment was calculated by dividing the enrichment of ChIP+ by that of ChIP‐. (d–f) Drought tolerance of target genes was assessed by measuring electrolyte leakage (d), MDA content (e), and ROS levels (f). The target genes were transiently transformed into Shanxin poplar for 48 h, followed by treatment with 20% PEG6000 for 24 h, after which physiological parameters were measured. Empty pROKII was used as the negative control. Data are presented as means ± SD from three independent biological replicates. mRNA down ‘refers to genes that are down‐regulated with a fold change of 2.0 or greater’, while ‘mRNA up’ refers to genes that are up‐regulated with a fold change of 2.0 or greater. * indicates a significant difference at *P* < 0.05 compared with pROKII under normal or drought conditions.

ChIP‐seq analysis identified 7784 genes whose promoters are potential binding targets of PdbSCL1. A joint analysis of ChIP‐seq and RNA‐seq data revealed that PdbSCL1 regulates 445 of these genes through promoter binding (Figure 3a). Among these, 33 genes were found to be up‐regulated, while 412 genes exhibited down‐regulation (Figure 3a). To validate the regulatory role of PdbSCL1 on these genes, we selected 10 genes for further analysis. We amplified the truncated promoters containing ABRE, PCF, and NFY motifs using ChIP–PCR, and the results confirmed that these truncated promoters could indeed be amplified (Figure 3b). RT‐qPCR analysis indicated that PdbSCL1 differentially regulates these genes (Figure 3c), supporting the reliability of the joint analysis findings.

Furthermore, five genes Pda_00000120 (*PdbbZIP‐53*), Pda_00037452 (*PdbACR12*), Pda_00041285 (*PdbACD6*), Pda_00005158 (*PdbRAP2‐3*), and Pda_00036150 (*PdbRCF3‐like*) stress respond and growth‐related gene targeted by PdbSCL1 were randomly selected for investigation of their roles in drought tolerance. These genes were transiently overexpressed in Shanxin poplar plants, with empty pROKII constructs serving as controls. Under normal conditions, all plant types exhibited similar levels of electrolyte leakage, MDA, and ROS (Figure 3d–f). However, upon treatment with 20% PEG6000, the plants overexpressing the five target genes demonstrated significantly lower electrolyte leakage, MDA levels, and ROS compared with the control plants (Figure 3d–f). These findings suggest that the five genes targeted by PdbSCL1 confer drought tolerance.

### Drought stress induces the acetylation of PdbSCL1

**Figure 4:**
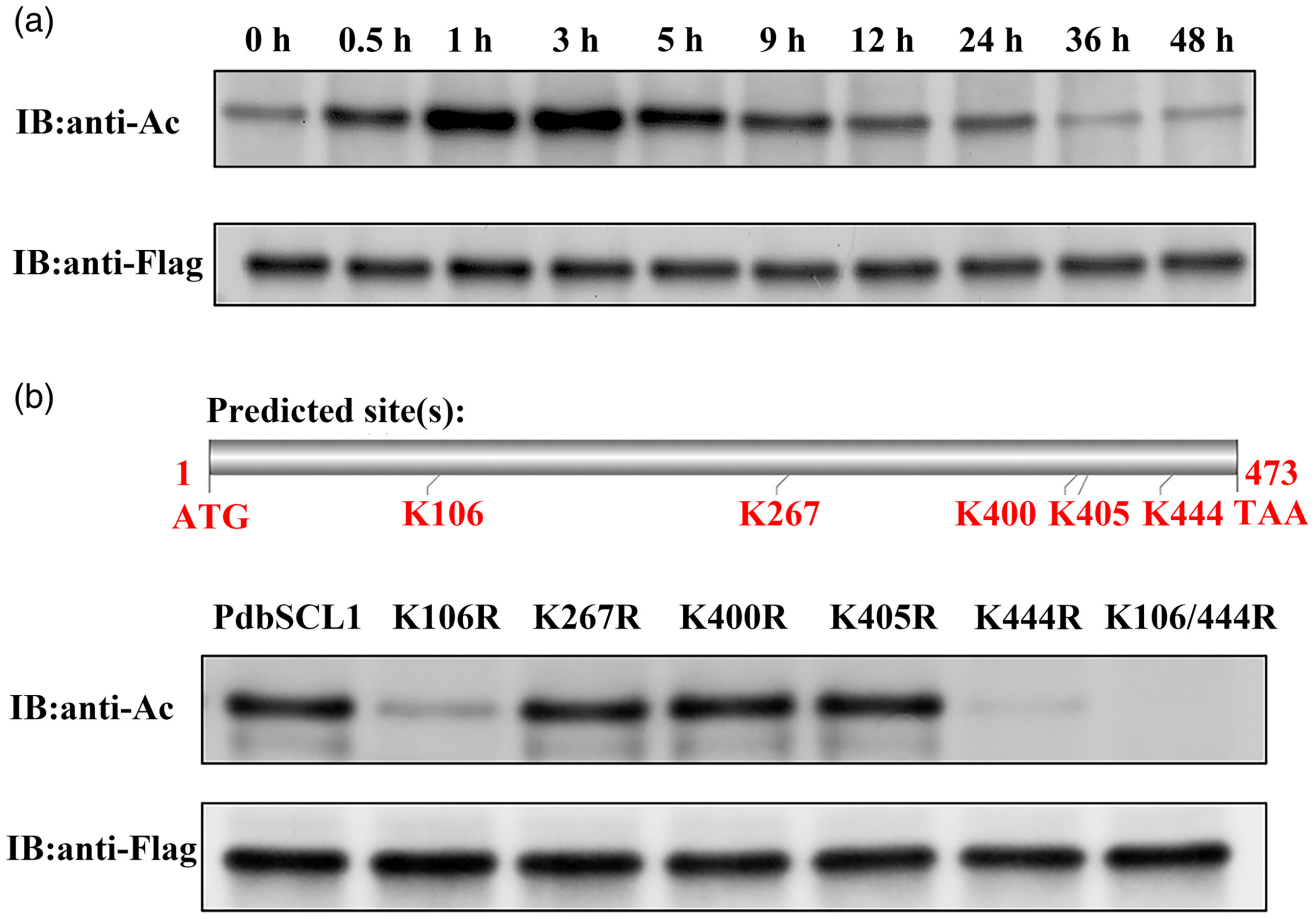
Detection of acetylation of PdbSCL1 protein. (a) Acetylation of PdbSCL1 was assessed under drought stress conditions. (b) Acetylation sites in PdbSCL1 were identified by mutating the potential acetylation site lysine (K) to arginine (R). The mutated genes, fused with a Flag‐tag, were transiently transformed into Shanxin poplar. PdbSCL1 was then immunoprecipitated using anti‐Flag antibody, and acetylation was analysed with an anti‐acetylation antibody. Anti‐FLAG was used as a control to detect protein levels, while anti‐Ac detected protein acetylation.

To investigate the acetylation of PdbSCL1 in response to drought stress, the PdbSCL1 protein was isolated from Shanxin poplar plants overexpressing PdbSCL1 using immunoprecipitation (IP) with an anti‐Flag antibody. The plants were subjected to drought stress induced by 20% PEG6000 for varying durations of 0, 1, 3, 5, 9, 12, 24, and 48 h. The acetylation of PdbSCL1 proteins was then assessed using an anti‐acetylation antibody. PdbSCL1 has a certain degree of acetylation at 0 h, which may be due to the presence of a baseline level of acetylation. The results indicated that as the treatment duration increased, the intensity of acetylated PdbSCL1 gradually increased, peaking at 9 h of drought stress before decreasing (Figure 4a). This finding suggests that drought stress induces the acetylation of PdbSCL1.

Subsequently, we aimed to identify the specific acetylation sites on PdbSCL1. Five potential acetylation sites were predicted (Figure 4b). These lysine residues (K) were individually mutated to arginine (R), and the resulting constructs were cloned into pROKII for overexpression. The mutated *PdbSCL1* genes were then transiently transformed into Shanxin poplar and subjected to PEG6000 treatment for 9 h. The acetylation status of the mutated PdbSCL1 proteins was analysed using Western blotting with the anti‐acetylation antibody. When lysine at positions 106 or 444 was replaced with arginine, the acetylation modification of PdbSCL1 was significantly reduced, while mutations at the other three sites did not affect acetylation. Additionally, simultaneous mutation of lysine to arginine at both positions 106 and 444 completely abolished acetylation. These results indicate that lysine residues 106 and 444 are critical sites for acetylation modification in PdbSCL1 (Figure 4b).

### Abolishing acetylation modification of PdbSCL1 impairs gene expression

To conduct a more comprehensive study on the role of acetylation modification in drought tolerance, we generated a double acetylation site mutation, PdbSCL1^K106/444R^ (where the lysines at positions 106 and 444 were both mutated to arginine), and stably transformed it into Shanxin poplar. The expression of PdbSCL1^K106/444R^ was analysed using RT‐qPCR (Figure S5), and the results confirmed successful transformation and expression in poplar. Three transgenic PdbSCL1^K106/444R^ lines with varying expression levels were selected for further investigation, designated as OX1, OX2, OX3.

**Figure 5:**
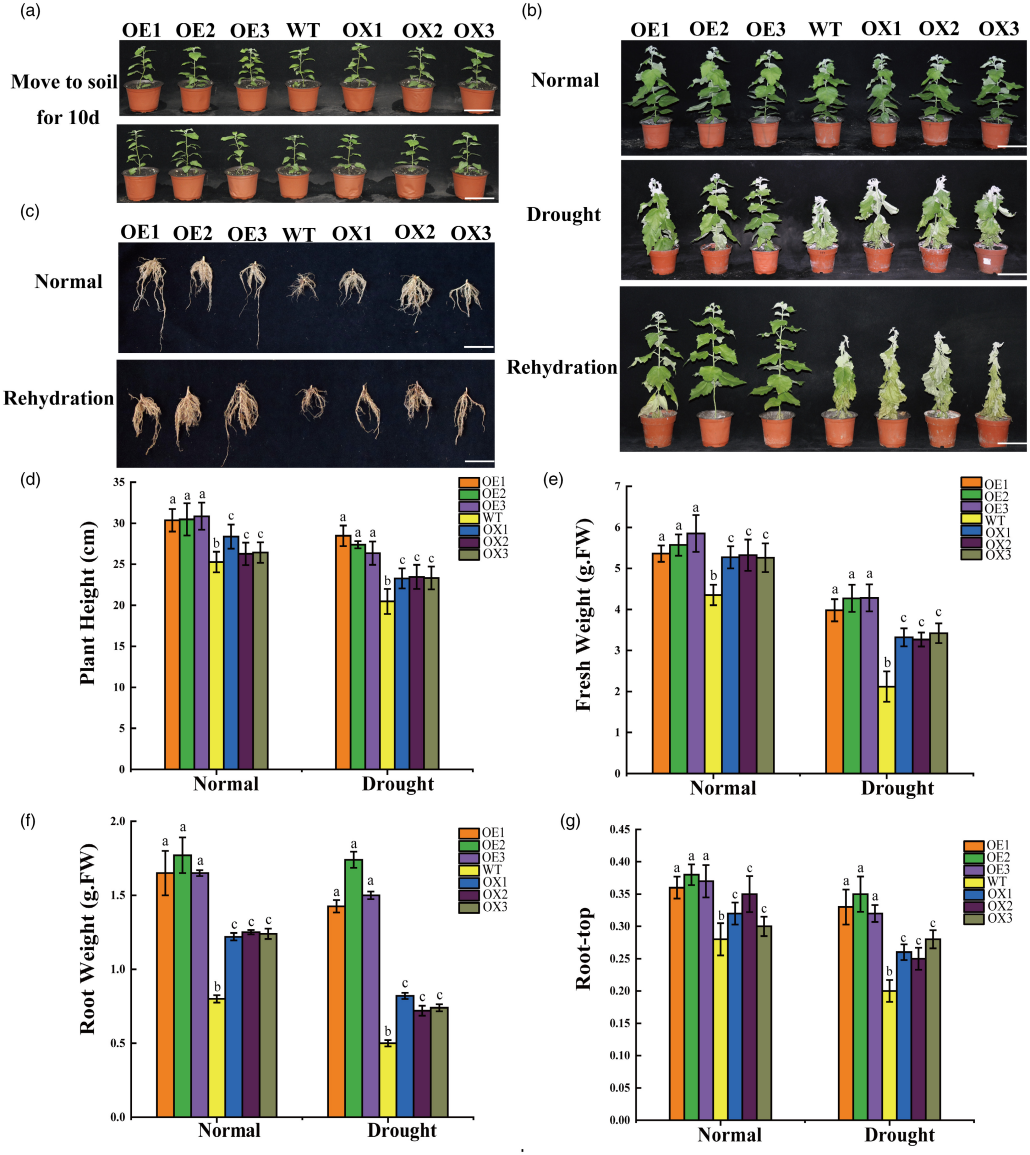
Comparison of drought tolerance between Shanxin poplar overexpressing *PdbSCL1* (OE) and PdbSCL1^K106/444R^ (OX). (a) Growth phenotypes of OE, WT, and OX lines immediately after transplantation into soil under control conditions. (b) Growth phenotypes of OE, WT, and OX lines under control, drought stress, and rehydration conditions. (c) Root growth of OE, WT, and OX lines under control and drought stress conditions. (d–g) Analysis of plant height (d), fresh weight (e), root fresh weight (f), and root‐to‐shoot ratio (g) of OE, WT, and OX lines. OE indicates Shanxin poplar lines overexpressing *PdbSCL1*; OX refers to lines with lysine (K) residues at positions 106 and 444 mutated to arginine (R), that is, PdbSCL1^K106/444R^. WT denotes wild‐type plants. ‘Normal’ indicates standard growth conditions, ‘drought’ indicates a 10‐day water deprivation, and ‘rehydration’ indicates well‐watered plants for 10 days after drought. Error bars represent the standard deviation (SD) from three biological replicates, each containing 5–10 plantlets.

These transgenic lines, which were of similar size, were transplanted into pots with soil (Figure 5a). After a period of growth, these plants were subjected to drought conditions by restricting water supply, with well‐watered plants serving as controls. After 20 days of drought stress followed by a 10‐day recovery period, the OE lines survived, while the WT and PdbSCL1^K106/444R^ lines perished (Figure 5b). Additionally, the OE lines exhibited the highest plant height, fresh weight, root weight, and root‐to‐shoot ratio, whereas OX1, OX2, OX3, and WT plants showed similar physiological traits under both normal and drought stress conditions (Figure 5c–g). These results indicate that abolishing acetylation of PdbSCL1 completely impairs its ability to confer drought tolerance.

We further compared the levels of MDA content, electrolyte leakage rate, SOD activity, POD activity, ROS content, and proline content. Under normal growth conditions, these physiological traits were similar among the lines. However, under drought conditions, OE lines exhibited significantly higher POD and SOD activity, as well as proline content, while WT and PdbSCL1^K106/444R^ lines showed comparably lower POD and SOD activity and proline levels. Additionally, OE lines had the lowest MDA content, electrolyte leakage rate, and ROS content, while WT and PdbSCL1^K106/444R^ lines were similar in these traits (Figure S6). These findings suggest that the loss of acetylation significantly diminishes the ability of PdbSCL1K to mediate drought tolerance, highlighting the essential role of acetylation modification.

### Abolishing acetylation does not affect subcellular localization but does impact transcriptional activation

**Figure 6:**
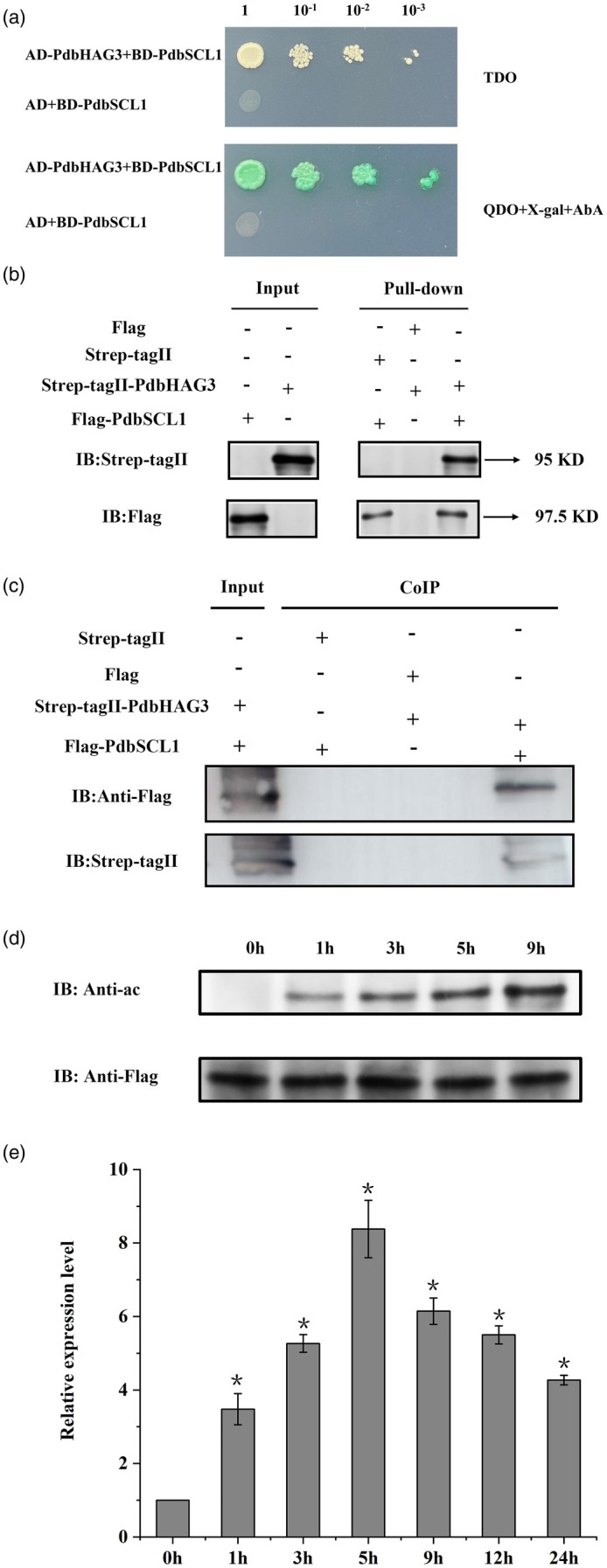
Identification of PdbSCL1 interacting proteins and its function. (a) Yeast two‐hybrid (Y2H) screening. This screening assessed the interaction between PdbSCL1 and PdbHAG3. TDO refers to synthetic dextrose (SD) /‐Leu/‐Trp‐His triple dropout medium. QDO/X/A indicates SD medium with ‐Leu/‐Trp/‐Ade/‐His quadruple dropout, supplemented with X‐α‐gal and Aureobasidin A (AbA) antibiotic. (b) Pull‐down analysis. The interaction between PdbSCL1 and PdbHAG3 was analysed using pull‐down assays. PdbHAG3 was fused with a Flag‐tag (PdbHAG3 + Flag), and PdbSCL1 was fused with a Strep‐tagII (PdbSCL1 + Strep‐tagII). Both proteins were expressed and purified from prokaryotic systems. The purified proteins were incubated together, followed by immunoprecipitation using anti‐Flag and anti‐Strep‐tagII antibodies. Western blot analysis was then conducted to detect the interaction partners, using anti‐Strep‐tagII antibody for products immunoprecipitated by anti‐Flag antibody, and anti‐Flag antibody for products isolated by anti‐Strep‐tagII antibody. ‘+’ indicates the addition of the reagent, while ‘‐’ indicates its absence. (c) Co‐immunoprecipitation (Co‐IP) confirmation of the interaction between PdbHAG3 and PdbSCL1. PdbHAG3 + Flag and PdbSCL1 + Strep‐tagII were cloned into the plant expression vector pROKII and transiently transformed into Shanxin poplar. For the input analysis, Western blotting was performed using anti‐Flag and anti‐Strep‐tagII antibodies, confirming the expression of both constructs in Shanxin poplar. For Co‐IP, the transformed plants were immunoprecipitated with anti‐Flag or anti‐Strep‐tagII antibodies, followed by Western blotting. The products of immunoprecipitation were detected using anti‐Flag antibody for those pulled down with anti‐Strep‐tagII antibody and vice versa. (d) Acetylation analysis: PdbHAG3 and PdbSCL1 were separately expressed and purified from prokaryotic systems, then incubated together for varying times. Acetylation analysis was conducted using an acetylated antibody. (e) Determination of the expression of PdbHAG3 in response to drought stress using qRT‐PCR. Asterisk (*) indicates statistical significance (*P* < 0.05).

Previous studies have indicated that acetylation modifications may affect the subcellular localization of proteins. Therefore, we investigated the subcellular localization of PdbSCL1 and PdbSCL1^K106/444R^. The results demonstrated that both proteins are located in the nucleus (Figure 6a), suggesting that acetylation modifications of PdbSCL1 do not influence its subcellular localization. To further examine the localization of PdbSCL1 in the cytoplasm and nucleus, we conducted Western blot analyses. Proteins from the cytoplasm and nucleus were isolated separately and subjected to Western blotting. Phosphoenolpyruvate carboxylase (PEPC) and Histone 3 served as markers for the cytoplasm and nucleus, respectively, while α‐tubulin was used as an internal reference. The results indicated that the GFP protein is present in both the cytoplasm and nucleus, while PdbSCL1 and PdbSCL1^K106/444R^ are exclusively located in the nucleus, further confirming our initial findings with GFP (Figure 6b).

To further investigate whether acetylation modification of PdbSCL1 affects gene expression, we examined three motifs bound by PdbSCL1 (ABRE, PCF, and NFY) that drive a GUS reporter gene, using PdbSCL1 and PdbSCL1^K106/444R^ as effectors. The 35S:Luc plasmid was co‐transformed for normalizing transformation efficiency. After transient co‐transformation of the reporter and effectors, the transformed plants were divided into two groups: one group was treated with PEG6000 for 9 h, while the other served as a control. The results showed that PEG treatment significantly induced GUS activity, whereas GUS activity without PEG treatment was comparable to that of the control (Figure 6c). Given that PdbSCL1 displayed acetylation modification while PdbSCL1^K106/444R^ lacked it (Figure 6d), these findings confirm that acetylation modification regulates gene expression mediated by PdbSCL1.

To further study how the loss of acetylation modification affects the regulation of PdbSCL1, we compared the expression of PdbSCL1 target genes between OE and PdbSCL1^K106/444R^ lines using RT‐qPCR. We randomly selected five target genes based on RNA‐seq data:(*PdbbZIP‐53*, *PdbACR12*, *PdbACD6*, *PdbRAP2‐3*, *PdbRCF3‐like*). The results revealed that compared with WT lines, the expression of these genes was only induced in the PdbSCL1‐OE lines, while no induction was observed in PdbSCL1^K106/444R^ lines (Figure 6e), indicating that acetylation modification is necessary for the regulation of gene expression mediated by PdbSCL1.

### The acetyltransferase PdbHAG3 interacts with PdbSCL1 and catalyses its acetylation in response to stress

**Figure 7:**
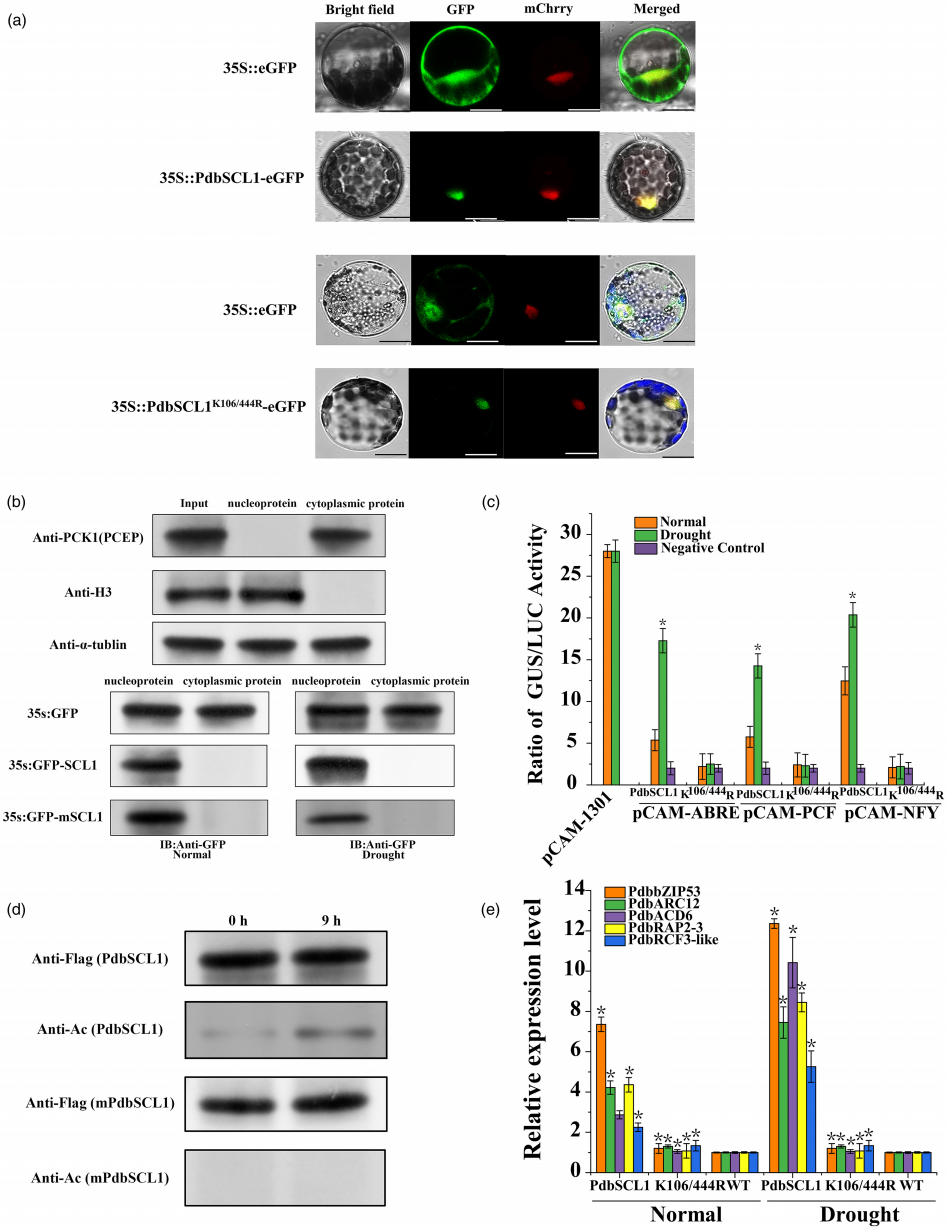
Effect of abolishing acetylation modification of PdbSCL1 on subcellular localization and element binding. (a) Subcellular localization analysis: The locations of PdbSCL1 and PdbSCL1^K106/444R^ were examined in *Arabidopsis thaliana* protoplasts. Green indicates eGFP fluorescence, while red indicates H2A‐mCherry, which marks nuclear localization. Merged images of bright‐field and eGFP are shown. Scale bar = 10 μm. (b) Western blot confirmation of subcellular location: Cytoplasmic and nuclear proteins of PdbSCL1 and PdbSCL1^K106/444R^ were isolated for Western blotting. Anti‐PCK1 antibody was used to check cytoplasmic contamination, and anti‐H3 antibody assessed nuclear protein quality. PdbSCL1 and PdbSCL1^K106/444R^ localization was analysed using anti‐GFP antibody. (c) Comparison of the transcription of PdbSCL1^K106/444R^ and PdbSCL1 on the DNA motifs ABRE, PCF, and NFY. The constructs 35S:PdbSCL1^K106/444R^ and 35S:PdbSCL1 served as effectors. Each motif was fused with a 35S minimal promoter to drive GUS expression. Additionally, 35S:Luc was co‐transformed into Shanxin poplar for normalization of transformation efficiency. GUS to Luc ratios were measured, with ‘Normal’ indicating standard conditions and ‘Drought’ indicating treatment with 20% PEG6000 for 9 h. (d) Acetylation modification analysis: PdbSCL1 and PdbSCL1^K106/444R^ were transiently expressed in plants and subjected to 20% PEG6000 treatment for 0 and 9 h. Immunoprecipitation was performed using anti‐Flag antibody, followed by acetylation analysis with an acetylated antibody. Three independent experiments were conducted, with error bars representing standard deviation (SD). *: An asterisk indicating significant differences (*P* < 0.05) from the control. (e) Comparison of the regulation of target genes betweenPdbSCL1^K106/444R^ and PdbSCL1. Expression of target genes was compared between plants overexpressing PdbSCL1^K106/444R^ and PdbSCL1 using qRT‐PCR.

Since PdbSCL1 can undergo acetylation, we sought to identify acetyltransferase proteins that interact with PdbSCL1. We retrieved 20 acetyltransferase genes from the genomes of Shanxin poplar and investigated their interaction with PdbSCL1 using Y2H assays. Y2H screening revealed a strong interaction between PdbSCL1 and PdbHAG3 (Figure 7a). To further confirm this interaction, we conducted pull‐down assays and co‐immunoprecipitation (Co‐IP), both of which confirmed that PdbSCL1 interacts with PdbHAG3 (Figure 7b,c). We also explored whether their interaction facilitates the acetylation of PdbSCL1K. Both PdbSCL1 and PdbHAG3 were expressed in prokaryotic systems; after purification, the proteins were incubated together for acetylation detection. The results indicated that the incubation of PdbSCL1 and PdbHAG3 led to acetylation, and as incubation time increased, the level of acetylation continued to rise (Figure 7d). In addition, the expression of PdbHAG3 is induced by drought stress (Figure 7e). These findings demonstrate that PdbHAG3 interacts with PdbSCL1 and facilitates the acetylation of PdbSCL1.

## Discussion

The GRAS family of TFs is critical for various aspects of plant growth and development (Neves *et al*., 2023; Jaiswal *et al*., 2022). Within this family, SCL proteins constitute a distinct subclass of GRAS TFs. While their functional significance is recognized, research on the role of PTMs of SCL proteins in stress responses and growth remains limited, indicating a gap in our understanding of their regulatory mechanisms.

### PdbSCL1 should be a promising candidate gene for future applications in molecular breeding

Previous studies have demonstrated that SCL TFs play a role in the biosynthesis of volatile terpenes and the development of glandular trichomes in tomato (Yang *et al*., 2021), as well as in the regulation of plant growth (Golldack *et al*., 2013). In the present study, we found that PdbSCL1 significantly promotes plant growth, as evidenced by increased plant height, total fresh weight, root weight, and root‐to‐shoot ratio (Figure 1a,d,e). Additionally, PdbSCL1 regulates the gene *PdbRAP2‐3*, which is associated with plant growth (Figure 3c). Previous studies showed that RAP2 family are closely involved in growth. For instance, RAP2.12 modulates plant metabolism through oxygen sensing, influencing growth and development by regulating fermentation enzyme activity and energy metabolism (Paul *et al*., 2016). Furthermore, RAP2.11 enhances Arabidopsis growth under low potassium conditions by regulating the AtHAK5 transporter, thereby improving survival (Kim *et al*., 2012). *RAP2.6* and *RAP2.6 L* are crucial for early development and stress responses; notably, overexpression of RAP2.6 results in dwarfism and increased branching (Krishnaswamy *et al*., 2011). Therefore, the enhanced growth observed with PdbSCL1 overexpression is likely due to its regulation of *PdbRAP2‐3*. Concurrently, PdbSCL1 also improves drought tolerance (Figure 1b). Collectively, these findings indicate that PdbSCL1 is a promising candidate gene for cultivating plants with rapid growth and improved drought resistance.

### Acetylation modifications play a critical role in gene expression mediated by PdbSCL1

Protein acetylation is a critical PTM that significantly regulates various biological processes in plants. Recent studies have highlighted its role in modulating gene expression, enzyme activity, and protein interactions, all of which influence plant growth, development, and responses to environmental stresses (Kumar *et al*., 2021; Xia *et al*., 2022). Additionally, proteomic analyses have revealed a diverse array of acetylated proteins across different plant tissues, suggesting that protein acetylation is a widespread modification affecting numerous cellular pathways (Li *et al*., 2018; Xia *et al*., 2022). However, much of the existing research has focused primarily on histone acetylation – an epigenetic modification that regulates genes involved in various biological processes (Kumar *et al*., 2021). Both histone acetylation and deacetylation have been implicated in responses to abiotic and biotic stresses, including drought and salinity (Cui *et al*., 2023; Gimenez‐Ibanez *et al*., 2022; Li *et al*., 2022). Despite this, there is a scarcity of studies examining the acetylation and deacetylation of TFs in response to abiotic stress, as well as the mechanisms by which these modifications influence transcriptional activity.

In the present study, we demonstrate that the acetylation of PdbSCL1 is induced by drought stress (Figure 4a), indicating its involvement in drought response. Furthermore, the deacetylation of PdbSCL1 leads to a loss of its ability to regulate gene expression (Figure 6c,d), ultimately compromising drought tolerance (Figure 5b–g). These findings suggest that PdbSCL1 acetylation enhances its capacity to regulate target genes critical for mediating drought resistance. We also identified PdbHAG3 as an interacting partner that acetylates PdbSCL1 (Figure 7), indicating the presence of a PdbHAG3‐PdbSCL1 module that contributes to drought tolerance in Shanxin poplar. Importantly, our results suggest that PdbSCL1 acetylation also plays a role in plant growth, as deacetylation reduces its capacity to promote growth (Figure 5b–g). This phenomenon may share a similar mechanism to that observed in drought tolerance, where the deacetylation of PdbSCL1 impairs its ability to regulate genes associated with growth, thereby inhibiting plant development. Further investigation in this area is warranted.

### PdbSCL1 interacts with various DNA motifs to regulate gene expression

TFs are essential regulators of gene expression, and their binding to specific DNA sequences is a fundamental aspect of cellular function and development. TFs recognize and bind to specific DNA motifs in the promoter and enhancer regions of genes, a critical interaction for initiating or repressing the transcription of target genes. Therefore, identifying the DNA motifs bound by a specific TF is necessary to gain a deeper understanding of its function. However, there are few reports on the identification of DNA motifs bound by GRAS family members.

In the present study, we identified the DNA motifs potentially bound by PdbSCL1 and found that it may interact with various types of DNA motifs (Figure 2b–h). Specifically, PdbSCL1 can bind to ABRE, PCF motifs, and NFY motifs to regulate gene expression (Figure 2i). The ABRE is an ABA‐responsive element located upstream of ABA‐dependent stress‐responsive genes and is involved in abiotic stress responses (Narusaka *et al*., 2003). The NFY motif, characterized by the CCAAT sequence, is recognized by the NF‐Y proteins, which play significant roles in fine‐tuning development, growth, stress responses, and hormone signalling (Gnesutta *et al*., 2018; Myers and Holt III, 2018). The PCF motif, with the sequence ‘TGGGCC/T’ is bound by TEOSINTE BRANCHED 1/CYCLOIDEA/PROLIFERATING CELL FACTOR 1 (TCP) TFs, which regulate growth and development in various plant species (Trémousaygue *et al*., 2003; Wang *et al*., 2022a). Additionally, TCP TFs are also important in mediating responses to abiotic stresses such as salt and drought (Liu *et al*., 2024; Willig *et al*., 2022).

Collectively, the proteins that bind to ABRE, PCF, and NFY motifs are involved in plant growth, development, and abiotic stress responses. Therefore, the ability of PdbSCL1 to bind to these motifs suggests that it also plays a role in plant growth, development, and abiotic stress response, which is consistent with the findings of this study.

### The regulator network of PdbSCL1 in response to drought

**Figure 8:**
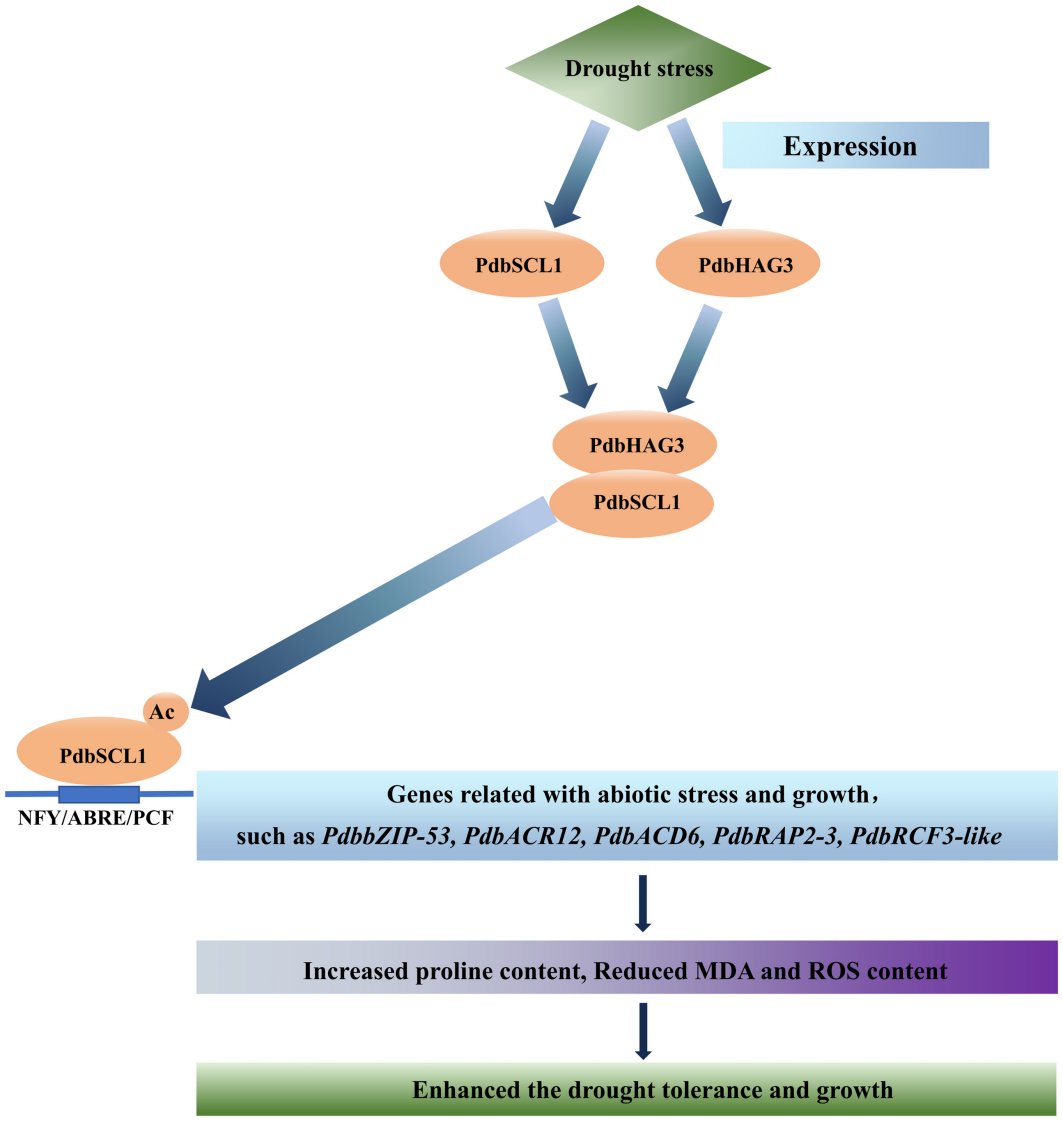
Working model of PdbSCL1 in modulating drought stress. Drought stress triggers the expression of PdbSCL1 and PdbHAG3, which then form a complex. This interaction leads to the acetylation of PdbSCL1. The acetylated PdbSCL1 subsequently binds to DNA motifs, such as ABRE, PCF, and NFY, which promote the expression of genes associated with drought stress and growth. The regulation of these genes results in physiological changes, including enhanced ROS scavenging capability and elevated proline, leading to reduced MDA content and decreased cell membrane damage. Ultimately, these alterations enhance the drought tolerance and growth of Shanxin poplar.

Building on the findings from the aforementioned studies, we propose a working model elucidating the role of PdbSCL1. Under normal conditions, PdbSCL1 has some extent of acetylation modification, which enables it to regulate some genes such as to facilitate growth. However, its acetylation is primarily induced by drought stress to enhance drought tolerance. Under drought conditions, both PdbSCL1 and PdbHAG3 are up‐regulated, leading to their interaction and the subsequent acetylation of PdbSCL1. The acetylated PdbSCL1 then activates the expression of a series of target genes by binding to the ABRE, PCF, and NFY motifs located in their promoters. This activation induces physiological changes that include a reduction in ROS accumulation and an increase in proline levels, which together lead to decreased electrolyte leakage and lower MDA levels, ultimately enhancing drought tolerance (Figure 8).

## Materials and methods

### Plant materials and treatments

Shanxin poplar (*Populus davidiana × P. bolleana*) tissue culture plantlets were grown on 1/2 MS solid medium with 0.5 mg/L 6‐benzylaminopurine (6‐BA) and 0.05 mg/L naphthaleneacetic acid (NAA) under a 12‐h light/12‐h dark cycle. After 2 weeks, the plantlets were transplanted into pots with a sterilized soil, vermiculite, and perlite mixture (4:2:1) and placed in a greenhouse with a 16‐h light/8‐h dark cycle, 70%–75% humidity, and 24°C temperature. To assess *PdbSCL1* expression under PEG6000 treatment, the plants were irrigated with 200 mL of a 20% (W/V) PEG6000 solution, and samples were collected after 1, 3, 5, 9, 12, and 24 h. Control plants were irrigated with fresh water. To assess drought tolerance, the plantlets were grown in a greenhouse with a relative humidity of ~50%, a photoperiod of 14 h of light and 10 h of darkness, and an average temperature of 25 °C. Plantlets of wild‐type (WT) and transgenic poplar, matched for size, were grown in soil in a greenhouse. Drought treatment involved withholding water for 10 days, followed by rehydration for 10 days, with well‐watered plants serving as controls.

### Phylogenetic tree construction and sequence alignment

The phylogenetic tree was generated using MEGA 6.0 through the neighbour‐joining (NJ) method, with 1000 bootstrap replicates. Multiple sequence alignment was performed using Clustal X 2.0 (Larkin *et al*., 2007; Tamura *et al*., 2013).

### RNA extraction, cDNA synthesis, and qRT‐PCR analysis

Total RNA from Shanxin poplar was isolated and reverse transcribed into cDNA using the PrimeScript™ RT reagent Kit. The cDNA was diluted to 100 μL for RT‐qPCR. The qPCR reaction mixture contained 10 μL SYBR Premix Ex Taq™ (Takara), 2 μL cDNA template, and 0.5 μM of both forward and reverse primers, for a total volume of 20 μL. qPCR was performed on a qTower 2.2 apparatus (Analytik Jena AG). The thermal cycling conditions include initial denaturation at 94 °C for 30 s, followed by 45 cycles of 94 °C for 12 s, 58 °C for 30 s, and 72 °C for 45 s. Tubulin was used as the internal control gene. Primers and GenBank accession numbers for all genes are listed in Table S1. Three biological replicates were performed, and relative expression levels were calculated using the $2^{\Delta \Delta C_{T}}$ method (Livak and Schmittgen, 2001).

### Generation of poplar plants with overexpressing and CRISPR edited *PdbSCL1*

The coding sequence (CDS) of *PdbSCL1* was in‐frame fused with 3 × Flag‐tags at the C‐terminal and cloned into the pROKII vector under the control of the 35S promoter. The gRNA (5′‐GAGAATCTTGACCTTGGAAATTTGCGG‐3′) sites, selected for high efficiency in Cas9 cleavage, were identified using the transient CRISPR/Cas editing method (Wang *et al*., 2021) and cloned into pEgP237‐2A‐GFP for CRISPR editing (Osakabe *et al*., 2016). All constructs were introduced into *Agrobacterium tumefaciens* strain EHA105 for transformation. Primers used for vector construction are listed in Table S1.

Stable genetic transformation of Shanxin poplar was achieved using the *Agrobacterium*‐mediated leaf disc method. Plantlet leaves were cut into explants and immersed for 5 min in a solution containing *A*. *tumefaciens* cells (OD_600_ = 0.3). The explants were then placed on co‐culture medium (WPM + 0.1 mg/L 6‐BA +2 mg/L 6‐BA +150 μM acetosyringone, pH 5.8) for 3 days. After co‐cultivation, the explants were transferred to a selection medium (WPM + 0.1 mg/L NAA + 2 mg/L 6‐BA +200 mg/L cephalosporin +200 mg/L tikarcillin +50 mg/L kanamycin for overexpression or 1 mg/L glyphosate for RNAi selection, pH 5.8) to promote adventitious bud development. Once the buds reached 3 cm, they were transferred to rooting medium (WPM + 0.25 mg/L NAA + 50 mg/L kanamycin or 1 mg/L glyphosate, pH 5.8) for rooting.

### Transient transformation

The transient transformation process was performed following the protocol established by Zang *et al*. (2015). Briefly, tissue culture plantlets of Shanxin poplar were utilized for the transformation. The seedlings were placed in a transformation solution consisting of 1/2 MS medium, 100 μM acetosyringone, 2.5% (w/v) sucrose, 0.01% (w/v) Tween 20, and *A*. *tumefaciens* at an OD_600_ of 0.8, adjusted to pH 5.6, and incubated at 25 °C while shaking at 90 rpm for 2.5 h. Following this, the poplar plants were rinsed twice with sterile distilled water and transferred to 1/2 MS solid medium (pH 5.8) containing 120 μm sugar and acetosyringone for 48 h.

### Physiological analysis

The levels of malondialdehyde (MDA), electrolyte leakage, superoxide dismutase (SOD), and peroxidase (POD) were assessed as outlined by Zang *et al*. (2015). Reactive oxygen species (ROS) were quantified using the plant reactive oxygen species (ROS) ELISA Detection Kit from Nanjing Senbeijia Bioengineering Institute in Nanjing, China. Proline content was measured according to the method described by Bates *et al*. (1973).

### RNA‐seq analysis

Poplar plants overexpressing PdbSCL1, along with WT Shanxin poplar, were subjected to a 20% PEG6000 solution for 9 h, followed by RNA‐seq analysis to compare gene expression between the two plant types. Three biological replicates were performed for accuracy. The RNA‐seq analysis was conducted by Wuhan Seqhealth Technology Co., Ltd. (Wuhan, China). Differentially expressed genes (DEGs) were identified using the criteria of an absolute log fold change (FC) > 1 and a *p*‐value ≤ 0.05.

### Yeast one‐hybrid (Y1H) and yeast two‐hybrid (Y2H) assay

PdbSCL1 was inserted into the pGADT7‐Rec vector, fused with the GAL4 activation domain, as the prey vector. The bait vector was constructed by cloning three tandem copies of motifs into the pHIS2 vector. Y1H assays were performed using the Match‐Maker™ One‐Hybrid System (Clontech; Mountain View, CA, USA) following the user manual. For yeast two‐hybrid (Y2H) analysis, PdbSCL1 was cloned into the pGBKT7 vector as the bait, and PdbHAG3 was inserted into the pGADT7‐Rec vector as the prey. Equal amounts of both plasmids were transformed into yeast Y2HGold cells (Clontech) and cultured on TDO (SD/‐His/‐Leu/‐Trp) and QDO (SD/‐Ade/‐His/‐Leu/‐Trp) media, supplemented with X‐a‐Gal (40 mg/L) and AbA (70 ng/mL), at 30 °C for 3–5 days. Co‐transformation of pGBKT7 with pGADT7‐Rec‐PdbHAG3 served as a negative control. All primers used are listed in Table S1.

### Electrophoretic mobility shift assay (EMSA)

The CDS of PdbSCL1 was cloned into the pMALc5X vector, which includes maltose‐binding protein (MBP), and expressed in *Escherichia coli* ER2523 (NEB, Ipswich, MA, USA). Recombinant protein purification was performed using the pMAL Protein Fusion & Purification System, following the provided protocols. Two oligo DNA sequences were biotin‐labelled at the 3' end for double‐stranded probe creation, with unlabeled probes as a control. The resulting complex was analysed by polyacrylamide gel electrophoresis (PAGE). The electrophoretic mobility shift assay (EMSA) was conducted using a chemiluminescent EMSA kit (Beyotime, Shanghai, China). Primers used are listed in Table S1.

### The transcriptional activation of PdbSCL1

The effector was constructed under the regulation of the 35S promoter in pROKII. For the reporter, three tandem copies of the ABA‐responsive element (ABRE, ‘CACGTG’), proliferating cell factors (PCF, ‘TGGGCC’), and nuclear factor‐Y (NF‐Y, ‘CCAAT’) core elements were linked to the 35S CaMV minimal promoter (−46 bp to +1) to drive expression of a GUS gene within a modified pCAMBIA1301 vector, from which the 35S:hygromycin cassette had been removed. Both the effector and reporter vectors were co‐transiently transformed into Shanxin poplar, along with the 35S::LUC construct to ensure normalization of transformation efficiency. The GUS and LUC activity ratios were calculated to assess the influence of PdbSCL1 on gene expression.

### Chromatin immunoprecipitation sequencing (ChIP‐seq) analysis

For ChIP analysis, transgenic plants expressing 35S:PdbSCL1‐Flag were used. The same RNA‐seq material was also used for ChIP‐seq. The ChIP procedure followed Zhao *et al*. (2020). Briefly, DNA and protein were crosslinked with 3% formaldehyde, and chromatin was sonicated into fragments ranging from 200 to 800 bp. Immunoprecipitation was performed using anti‐Flag antibody (Beyotime), and the samples were subjected to high‐throughput sequencing. Raw reads were processed to remove low‐quality and short reads, then aligned to the Shanxin genome using Bowtie2 with default settings. Enriched regions (peaks) were identified using MACS 1.4 (Zhang *et al*., 2008). Peak analysis was performed with PeakAnalyzer (Salmon‐Divon *et al*., 2010), and the PeakAnnotator function was used to identify functional elements near peak loci via the nearest downstream gene (NDG) subroutine.

### Western blot analysis

The proteins were separated by SDS‐PAGE and transferred to a PVDF membrane (Millipore). To reduce non‐specific binding, the membrane was blocked using the Quick Block™ Western kit (Beyotime). It was then incubated overnight at 4 °C with anti‐Flag antibody. HRP‐conjugated secondary antibodies were applied, and enhanced chemiluminescence (ECL) solution (Beyotime) was used for detection. Signals were captured using ECL Prime (GE Healthcare; Delaware, Chicago, USA).

### Analysis of PdbSCL1 acetylation

Poplar plants overexpressing *PdbSCL1* were treated with 20% PEG6000 for 1, 3, 5, and 9 h, then collected. Proteins were extracted using a Plant Western and IP cell lysate kit (Beyotime) and immunoprecipitated with anti‐Flag antibody. The sample was divided into two portions: one was analysed by Western blotting with anti‐Flag antibody, and the other was assessed with an anti‐acetylation antibody (ICP0380, IMMUNECHEM, Canada). SDS‐PAGE was used to separate proteins, which were transferred to PVDF membranes (Millipore). Membranes were blocked with 5% skim milk, then incubated with anti‐acetylation or anti‐Flag antibodies. HRP‐conjugated secondary antibodies were applied, and detection was performed using ECL solution (Beyotime). Signals were captured with ECL Prime (GE Healthcare).

### Determination of the acetylation site of PdbSCL1

Potential acetylation lysine sites were predicted using GPS‐PAIL 2.0. Mutagenesis at five sites was performed with the Quick Mutation™ Plus kit (Beyotime), substituting lysine (K) with arginine (R) (D0208S). Each mutated *PdbSCL1* sequence, tagged with 3xFlag, was cloned into the pROKII vector and transiently transformed into Shanxin poplar. After 48 h, the plants were treated with 20% PEG6000 for 9 h. Proteins were extracted, immunoprecipitated with anti‐Flag antibody, and separated by SDS‐PAGE. Acetylation levels were assessed by Western blotting using the Ace‐lys antibody.

### Co‐immunoprecipitation (Co‐IP) analysis

*PdbSCL1* and *PdbHAG3* were individually fused with Flag‐tag and Strep‐tagII at their C‐termini, then cloned into the pROKII vector. Additionally, 35S:Flag and 35S:Strep‐tagII constructs were also cloned into pROKII, resulting in four constructs in total. These constructs were paired and transiently transformed into WT Shanxin poplar plants. After extracting nuclear proteins, the samples were divided into two aliquots. One aliquot was immunoprecipitated with anti‐Flag antibody, and the other with anti‐Strep‐tagII antibody (ab119820; Abcam, UK). For Western blotting, the anti‐Flag‐immunoprecipitated sample was analysed with anti‐Strep‐tagII antibody, and the anti‐Strep‐tagII‐immunoprecipitated sample was analysed with anti‐Flag antibody.

### Pull‐down assays

PdbSCL1 and PdbHAG3 were fused with Flag‐tag and Strep‐tagII at their C‐termini, then cloned into the pMAL‐c5X vector for expression in *E*. *coli* ER2523. Different protein combinations were mixed in equal amounts with 1× binding buffer and isolated using either Flag or Strep‐tagII beads. Western blotting was performed using Flag and Strep‐tagII antibodies to analyse the proteins. To assess acetylation of PdbSCL1 by PdbHAG3, a mixture of 100 μL purified PdbSCL‐Flag and PdbHAG3‐Strep‐tagII proteins in binding buffer (50 mm Tris–HCl, pH 7.5; 100 mm NaCl; 0.25% Triton‐X100; 35 mm DTT) was incubated at 4 °C for 2 h. Acetylation of PdbSCL1 was detected by Western blotting.

### Statistical analyses

The software program SPSS v16.0 (IBM Corp., Armonk, NY, USA) was used to conduct the statistical analyses. The multiple comparisons were performed using one‐way ANOVA in SPSS, with the comparison method being the least significant difference (LSD) test. The statistical significance level for the LSD test used to compare the source data was set at *P* < 0.05.

## Author contributions

Yucheng Wang and Dandan Li designed the study; Pengyu Wang and Xue Yang performed the research; Pengyu Wang, Xue Yang, Shilin Sun, Jingxin Wang, Jingwen Wang, and Xiaofu Li analysed the data; Yucheng Wang, Dandan Li, and Pengyu Wang wrote the article.

## Conflict of interest

The authors declare no conflict of interest.

## Supporting information


**Figure S1** Expression and bioinformatic analysis of PdbSCL1. Phylogenetic analysis of PdbSCL1 protein and GRAS transcription factor protein in *Arabidopsis thaliana* and *Populus pilocarpa* by MEGA6 neighbour‐joining method. (B) Multiple sequences analysis of GRAS proteins. *A. thaliana* (ATSCL3), *P. trichocarpa* (PtrGRAS1, PtrGRAS56). (C)The expression profiles of PdbSCL1 in response to PEG6000 treatment using RT‐qPCR. Plants were treated with 20%(W/V)PEG6000 for 0 (control), 1 3, 5, 9, 12, and 24 h. The expression of *PdbSCL1* at 0 h was set as 1 to calculate the relative expression of *PdbSCL1* at different stress time points. The error bar represents the standard deviation (SD) of three biological replicates. Asterisks indicate statistically signiffcant differences between 0 h and other times (*P < 0.05).

**Figure S2** Generation *Populus davidiana × P. bolleana* of lines with knockout, and overexpression of PdbSCL1. (A) PCR was used to detect whether PdbSCL1 was inserted into the genome. (B) RT‐qPCR analyses the expression of *PdbSCL1* in different overexpression lines (OE). WT was used as a control and set to 1. The error bar represents the standard deviation (SD) of three biological replicates. Asterisks indicate a significant difference at *P* < 0.05. (C) Western blot was used to detect whether the gene is translated and expresses the correct protein. (D) Analysis of the mutation of PdbSCL1 (*scl*) induced by CRISPR using Sanger DNA sequencing. The target sequence and insertion nucleotides had been indicated.

**Figure S3** Determination of physiological parameters involved in drought stress tolerance. (A) Measurement of MDA content. (B) Electrolyte leakage rates. (C) SOD activity. (D) POD activity. (E) ROS content. (F) Proline content. Data are presented as means ± SD from three independent experiments. Asterisks (*) indicate significant differences (*P* < 0.05) compared with the control under normal or drought conditions. OE, overexpressing *PdbSCL1* Shanxin poplar lines; *scl*, Shanxin poplar with *PdbSCL1* knockout induced by CRISPR/Cas; WT, wild‐type plants. ‘Normal’ indicates plants grown under standard conditions; ‘drought’ indicates plants treated with no water for 10 days.

**Figure S4** Differentially expressed genes of PdbSCL1 followed by RNA‐seq analysis. The column diagram illustrates the number of differentially expressed genes (DEGs) between the PdbSCL1‐OE lines and the wild‐type (WT) following exposure to a 20% PEG6000 solution for 9 h.

**Figure S5** RT‐qPCR analyses the expression of PdbSCL1^K106/444R^ in different overexpression lines (OX). WT was used as a control and set to 1. The error bar represents the standard deviation (SD) of three biological replicates. Asterisks indicate a significant difference at *P* < 0.05.

**Figure S6** Comparison of physiological parameters related to drought stress tolerance between PdbSCL1 and PdbSCL1^K106/444R^ with mutated acetylation sites. (A) Measurement of MDA content. (B) Analysis of electrolyte leakage rates. (C) Assessment of SOD activity. (D) Measurement of POD activity. (E) Determination of ROS content. (F) Measurement of proline content. Data are presented as means ± SD from three independent experiments. Asterisks (*) indicate significant differences (*t*‐test, *P* < 0.05) compared with the control under normal or drought conditions. OE, Shanxin poplar lines overexpressing PdbSCL1; K106/444R1‐3, Shanxin poplar overexpressing PdbSCL1^K106/444R^; WT, wild‐type plants. ‘Normal’ indicates plants were grown under normal conditions; ‘drought’ refers to plants that were treated without water for a duration of 10 days.

**Table S1** Primers used in this study.

**Table S2** Differentially expressed genes of PdbSCL1 overexpression (OE) and WT plants after subjected to a 20% PEG6000 solution for 9 h.

## Data Availability

Sequence data from this article can be found in the NCBI database under the following accession numbers: PdbbZIP‐53 (PQ465176), PdbACR12 (PQ465177), Unknownprotein (PQ465178, PdbACD6 (PQ465179), PdbKL(PQ465180)), dormancy‐associated protein homolog 4 (PQ465181), PdbRAP2‐3 (PQ465182), Hydrolase (PQ465183), Leucine‐rich protein (PQ465184), PdbRCF3‐like (PQ465185), Tubulin (PQ465186), PdbSCL1 (PQ465187), PdbHAG3 (PQ465188), PdbSCL1 overexpression (OE) and WT plants after 10 days of drought stress row data stored in NCBI with submission number NCBI GEO: PRJNA1134423 xxxFF0C;To identify the binding regions and the genes directly regulated by PdbSCL1, we performed ChIP‐seq analysis row data stored in NCBI with submission number: PRJNA1131963.

## References

[pbi70185-bib-0001] Bates, L.S. , Waldren, R. and Teare, I. (1973) Rapid determination of free proline for water‐stress studies. Plant Soil, 39, 205–207.

[pbi70185-bib-0002] Bisht, A. , Eekhout, T. , Canher, B. , Lu, R. , Vercauteren, I. , De Jaeger, G. , Heyman, J. *et al*. (2023) PAT1‐type GRAS‐domain proteins control regeneration by activating DOF3. 4 to drive cell proliferation in Arabidopsis roots. Plant Cell, 35, 1513–1531. 36747478 10.1093/plcell/koad028 PMC10118276

[pbi70185-bib-0003] Cai, H. , Chen, Y. , Zhang, M. , Cai, R. , Cheng, B. , Ma, Q. and Zhao, Y. (2017) A novel GRAS transcription factor, ZmGRAS20, regulates starch biosynthesis in rice endosperm. Physiol. Mol. Biol. Plants, 23, 143–154. 28250591 10.1007/s12298-016-0404-9 PMC5313408

[pbi70185-bib-0005] Cui, X. , Dard, A. , Reichheld, J.‐P. and Zhou, D.‐X. (2023) Multifaceted functions of histone deacetylases in stress response. Trends Plant Sci. 28, 1245–1256. 37394308 10.1016/j.tplants.2023.06.006

[pbi70185-bib-0006] Dutta, M. , Saha, A. , Moin, M. and Kirti, P.B. (2021) Genome‐wide identification, transcript profiling and bioinformatic analyses of GRAS transcription factor genes in rice. Front. Plant Sci. 12, 777285. 34899804 10.3389/fpls.2021.777285 PMC8660974

[pbi70185-bib-0007] Gimenez‐Ibanez, S. , Espinosa‐Cores, L. and Solano, R. (2022) Reversible acetylation fine‐tunes plant hormone signaling and immunity. Mol. Plant, 15, 1415–1417. 35927952 10.1016/j.molp.2022.07.015

[pbi70185-bib-0008] Gnesutta, N. , Mantovani, R. and Fornara, F. (2018) Plant flowering: imposing DNA specificity on histone‐fold subunits. Trends Plant Sci. 23, 293–301. 29331540 10.1016/j.tplants.2017.12.005

[pbi70185-bib-0009] Golldack, D. , Li, C. , Mohan, H. and Probst, N. (2013) Gibberellins and abscisic acid signal crosstalk: living and developing under unfavorable conditions. Plant Cell Rep. 32, 1007–1016. 23525744 10.1007/s00299-013-1409-2

[pbi70185-bib-0010] Gong, F. , Yu, W. , Cao, K. , Xu, H. and Zhou, X. (2024) RcTRP5 transcription factor mediates the molecular mechanism of lignin biosynthesis regulation in *R. chrysanthum* against UV‐B stress. Int. J. Mol. Sci. 25, 9205. 39273154 10.3390/ijms25179205 PMC11395560

[pbi70185-bib-0011] He, Z. , Tian, Z. , Zhang, Q. , Wang, Z. , Huang, R. , Xu, X. , Wang, Y. *et al*. (2022) Genome‐wide identification, expression and salt stress tolerance analysis of the GRAS transcription factor family in *Betula platyphylla* . Front. Plant Sci. 13, 1022076. 36352865 10.3389/fpls.2022.1022076 PMC9638169

[pbi70185-bib-0012] Heo, J.‐O. , Chang, K.S. , Kim, I.A. , Lee, M.‐H. , Lee, S.A. , Song, S.‐K. , Lee, M.M. *et al*. (2011) Funneling of gibberellin signaling by the GRAS transcription regulator scarecrow‐like 3 in the Arabidopsis root. Proc. Natl Acad. Sci. USA, 108, 2166–2171. 21245304 10.1073/pnas.1012215108 PMC3033297

[pbi70185-bib-0013] Hirano, Y. , Nakagawa, M. , Suyama, T. , Murase, K. , Shirakawa, M. , Takayama, S. , Sun, T.‐p. *et al*. (2017) Structure of the SHR–SCR heterodimer bound to the BIRD/IDD transcriptional factor JKD. Nat. Plants, 3, 1–10. 10.1038/nplants.2017.10 PMC5639936 28211915

[pbi70185-bib-0014] Hofmann, N.R. (2016) A structure for plant‐specific transcription factors: the GRAS domain revealed. Plant Cell 28, 993–994. 27095838 10.1105/tpc.16.00309 PMC4904681

[pbi70185-bib-0015] Jaiswal, V. , Kakkar, M. , Kumari, P. , Zinta, G. , Gahlaut, V. and Kumar, S. (2022) Multifaceted roles of GRAS transcription factors in growth and stress responses in plants. iScience, 25, 105026. 36117995 10.1016/j.isci.2022.105026 PMC9474926

[pbi70185-bib-0016] Kim, M.J. , Ruzicka, D. , Shin, R. and Schachtman, P. (2012) The Arabidopsis AP2/ERF transcription factor RAP2. 11 modulates plant response to low‐potassium conditions. Mol. Plant 5, 1042–1057. 22406475 10.1093/mp/sss003

[pbi70185-bib-0017] Krishnaswamy, S. , Verma, S. , Rahman, M.H. and Kav, N.N. (2011) Functional characterization of four APETALA2‐family genes (*RAP2. 6*, *RAP2. 6L*, *DREB19* and *DREB26*) in Arabidopsis. Plant Mol. Biol. 75, 107–127. 21069430 10.1007/s11103-010-9711-7

[pbi70185-bib-0018] Kumar, V. , Thakur, J.K. and Prasad, M. (2021) Histone acetylation dynamics regulating plant development and stress responses. Cell. Mol. Life Sci. 78, 4467–4486. 33638653 10.1007/s00018-021-03794-x PMC11072255

[pbi70185-bib-0019] Larkin, M.A. , Blackshields, G. , Brown, N.P. , Chenna, R. , McGettigan, P.A. , McWilliam, H. , Valentin, F. *et al*. (2007) Clustal W and Clustal X version 2.0. Bioinformatics, 23, 2947–2948. 17846036 10.1093/bioinformatics/btm404

[pbi70185-bib-0020] Li, X. , Ye, J. , Ma, H. and Lu, P. (2018) Proteomic analysis of lysine acetylation provides strong evidence for involvement of acetylated proteins in plant meiosis and tapetum function. Plant J. 93, 142–154. 29124795 10.1111/tpj.13766

[pbi70185-bib-0021] Li, Z. , Zhang, H. , Cai, C. , Lin, Z. , Zhen, Z. , Chu, J. and Guo, K. (2022) Histone acetyltransferase GCN5‐mediated lysine acetylation modulates salt stress aadaption of Trichoderma. Appl. Microbiol. Biotechnol. 106, 3033–3049. 35376971 10.1007/s00253-022-11897-z

[pbi70185-bib-0022] Li, Y. , Zhang, R. and Hei, H. (2023) Advances in post‐translational modifications of proteins and cancer immunotherapy. Front. Immunol. 14, 1229397. 37675097 10.3389/fimmu.2023.1229397 PMC10477431

[pbi70185-bib-0023] Liu, Z. , Shi, X. , Wang, Z. , Qu, M. , Gao, C. , Wang, C. and Wang, Y. (2024) Acetylation of transcription factor BpTCP20 by acetyltransferase BpPDCE23 modulates salt tolerance in birch. Plant Physiol. 195, 2354–2371. 38501602 10.1093/plphys/kiae168

[pbi70185-bib-0024] Livak, K.J. and Schmittgen, T.D. (2001) Analysis of relative gene expression data using real‐time quantitative PCR and the 2^−ΔΔCT^ method. Methods, 25, 402–408. 11846609 10.1006/meth.2001.1262

[pbi70185-bib-0025] Morohashi, K. , Minami, M. , Takase, H. , Hotta, Y. and Hiratsuka, K. (2003) Isolation and characterization of a novel GRAS gene that regulates meiosis‐associated gene expression. J. Biol. Chem. 278, 20865–20873. 12657631 10.1074/jbc.M301712200

[pbi70185-bib-0026] Myers, Z.A. and Holt, B.F., III (2018) NUCLEAR FACTOR‐Y: still complex after all these years? Curr. Opin. Plant Biol. 45, 96–102. 29902675 10.1016/j.pbi.2018.05.015

[pbi70185-bib-0027] Narita, T. , Weinert, B.T. and Choudhary, C. (2019) Functions and mechanisms of non‐histone protein acetylation. Nat. Rev. Mol. Cell Biol. 20, 156–174. 30467427 10.1038/s41580-018-0081-3

[pbi70185-bib-0028] Narusaka, Y. , Nakashima, K. , Shinwari, Z.K. , Sakuma, Y. , Furihata, T. , Abe, H. , Narusaka, M. *et al*. (2003) Interaction between two cis‐acting elements, ABRE and DRE, in ABA‐dependent expression of Arabidopsis rd29A gene in response to dehydration and high‐salinity stresses. Plant J. 34, 137–148. 12694590 10.1046/j.1365-313x.2003.01708.x

[pbi70185-bib-0054] Neves, C. , Ribeiro, B. , Amaro, R. , Expósito, J. , Grimplet, J. and Fortes, A.M. (2023) Network of GRAS transcription factors in plant development, fruit ripening and stress responses. Hortic. Res. 10, uhad220. 38077496 10.1093/hr/uhad220 PMC10699852

[pbi70185-bib-0029] Osakabe, Y. , Watanabe, T. , Sugano, S. , Ueta, R. , Ishihara, R. , Shinozaki, K. and Osakabe, K. (2016) Optimization of CRISPR/Cas9 genome editing to modify abiotic stress responses in plants. Sci. Rep. 6, 26685. 27226176 10.1038/srep26685 PMC4880914

[pbi70185-bib-0030] Paul, M.V. , Iyer, S. , Amerhauser, C. , Lehmann, M. , van Dongen, J.T. and Geigenberger, P. (2016) Oxygen sensing via the ethylene response transcription factor RAP2. 12 affects plant metabolism and performance under both normoxia and hypoxia. Plant Physiol. 172, 141–153. 27372243 10.1104/pp.16.00460 PMC5074624

[pbi70185-bib-0031] Salmon‐Divon, M. , Dvinge, H. , Tammoja, K. and Bertone, P. (2010) PeakAnalyzer: genome‐wide annotation of chromatin binding and modification loci. BMC Bioinformatics, 11, 1–12. 20691053 10.1186/1471-2105-11-415 PMC2923140

[pbi70185-bib-0032] Shvedunova, M. and Akhtar, A. (2022) Modulation of cellular processes by histone and non‐histone protein acetylation. Nat. Rev. Mol. Cell Biol. 23, 329–349. 35042977 10.1038/s41580-021-00441-y

[pbi70185-bib-0033] Tamura, K. , Stecher, G. , Peterson, D. , Filipski, A. and Kumar, S. (2013) MEGA6: molecular evolutionary genetics analysis version 6.0. Mol. Biol. Evol. 30, 2725–2729. 24132122 10.1093/molbev/mst197 PMC3840312

[pbi70185-bib-0034] Torres‐Galea, P. , Hirtreiter, B. and Bolle, C. (2013) Two GRAS proteins, SCARECROW‐LIKE21 and PHYTOCHROME A SIGNAL TRANSDUCTION1, function cooperatively in phytochrome A signal transduction. Plant Physiol. 161, 291–304. 23109688 10.1104/pp.112.206607 PMC3532260

[pbi70185-bib-0035] Trémousaygue, D. , Garnier, L. , Bardet, C. , Dabos, P. , Hervé, C. and Lescure, B. (2003) Internal telomeric repeats and ‘TCP domain’ protein‐binding sites co‐operate to regulate gene expression in *Arabidopsis thaliana* cycling cells. Plant J. 33, 957–966. 12631321 10.1046/j.1365-313x.2003.01682.x

[pbi70185-bib-0036] Uhrig, R.G. , Schläpfer, P. , Roschitzki, B. , Hirsch‐Hoffmann, M. and Gruissem, W. (2019) Diurnal changes in concerted plant protein phosphorylation and acetylation in Arabidopsis organs and seedlings. Plant J. 99, 176–194. 30920011 10.1111/tpj.14315

[pbi70185-bib-0037] Wang, Z. , He, Z. , Qu, M. , Liu, Z. , Wang, C. and Wang, Y. (2021) A method for determining the cutting efficiency of the crispr/cas system in birch and poplar. For. Res. 1, 16. 10.48130/FR-2021-0016 PMC11524279 39524512

[pbi70185-bib-0038] Wang, Y. , Yu, Y. , Wan, H. , Tang, J. and Ni, Z. (2022a) The sea‐Island cotton GbTCP4 transcription factor positively regulates drought and salt stress responses. Plant Sci. 322, 111329. 35667469 10.1016/j.plantsci.2022.111329

[pbi70185-bib-0039] Wang, P. , Wang, J. , Sun, X. , Yang, X. , Sun, S. , Han, X. , Li, D. *et al*. (2022b) Construction of a hierarchical gene regulatory network to reveal the drought tolerance mechanism of Shanxin poplar. Int. J. Mol. Sci. 24, 384. 36613845 10.3390/ijms24010384 PMC9820611

[pbi70185-bib-0040] Waseem, M. , Nkurikiyimfura, O. , Niyitanga, S. , Jakada, B.H. , Shaheen, I. and Aslam, M.M. (2022) GRAS transcription factors emerging regulator in plants growth, development, and multiple stresses. Mol. Biol. Rep. 49, 9673–9685. 35713799 10.1007/s11033-022-07425-x

[pbi70185-bib-0041] Willig, J.J. , Guarneri, N. , van Steenbrugge, J.J. , de Jong, W. , Chen, J. , Goverse, A. , Lozano Torres, J.L. *et al*. (2022) The Arabidopsis transcription factor TCP9 modulates root architectural plasticity, reactive oxygen species‐mediated processes, and tolerance to cyst nematode infections. Plant J. 112, 1070–1083. 36181710 10.1111/tpj.15996 PMC9828446

[pbi70185-bib-0042] Xia, L. , Kong, X. , Song, H. , Han, Q. and Zhang, S. (2022) Advances in proteome‐wide analysis of plant lysine acetylation. Plant Commun. 3, 100266. 35059632 10.1016/j.xplc.2021.100266 PMC8760137

[pbi70185-bib-0043] Yang, C. , Marillonnet, S. and Tissier, A. (2021) The scarecrow‐like transcription factor SlSCL3 regulates volatile terpene biosynthesis and glandular trichome size in tomato (*Solanum lycopersicum*). Plant J. 107, 1102–1118. 34143914 10.1111/tpj.15371

[pbi70185-bib-0044] Yolcu, S. , Ozdemir, F. , Güler, A. and Bor, M. (2016) Histone acetylation influences the transcriptional activation of POX in *Beta vulgaris* L. and *Beta maritima* L. under salt stress. Plant Physiol. Biochem. 100, 37–46. 26773543 10.1016/j.plaphy.2015.12.019

[pbi70185-bib-0045] Yu, L. , Hui, C. , Huang, R. , Wang, D. , Fei, C. , Guo, C. and Zhang, J. (2023) Genome‐wide identification, evolution and transcriptome analysis of GRAS gene family in Chinese chestnut (*Castanea mollissima*). Front. Genet. 13, 1080759. 36685835 10.3389/fgene.2022.1080759 PMC9845266

[pbi70185-bib-0046] Zang, D. , Wang, C. , Ji, X. and Wang, Y. (2015) *Tamarix hispida* zinc finger protein ThZFP1 participates in salt and osmotic stress tolerance by increasing proline content and SOD and POD activities. Plant Sci. 235, 111–121. 25900571 10.1016/j.plantsci.2015.02.016

[pbi70185-bib-0047] Zhang, X. , Yuan, Z. , Zhang, Y. , Yong, S. , Salas‐Burgos, A. , Koomen, J. , Olashaw, N. *et al*. (2007) HDAC6 modulates cell motility by altering the acetylation level of cortactin. Mol. Cell, 27, 197–213. 17643370 10.1016/j.molcel.2007.05.033 PMC2684874

[pbi70185-bib-0048] Zhang, Y. , Liu, T. , Meyer, C.A. , Eeckhoute, J. , Johnson, D.S. , Bernstein, B.E. , Nusbaum, C. *et al*. (2008) Model‐based analysis of ChIP‐Seq (MACS). Genome Biol. 9, 1–9. 10.1186/gb-2008-9-9-r137 PMC2592715 18798982

[pbi70185-bib-0049] Zhang, B. , Su, L. , Hu, B. and Li, L. (2018) Expression of AhDREB1, an AP2/ERF transcription factor gene from peanut, is affected by histone acetylation and increases abscisic acid sensitivity and tolerance to osmotic stress in Arabidopsis. Int. J. Mol. Sci. 19, 1441. 29751673 10.3390/ijms19051441 PMC5983730

[pbi70185-bib-0050] Zhao, L. , Wang, P. , Yan, S. , Gao, F. , Li, H. , Hou, H. and Li, L. (2014) Promoter‐associated histone acetylation is involved in the osmotic stress‐induced transcriptional regulation of the maize ZmDREB2A gene. Physiol. Plant. 151, 459–467. 24299295 10.1111/ppl.12136

[pbi70185-bib-0051] Zhao, H. , Li, H. , Jia, Y. , Wen, X. , Guo, H. , Xu, H. and Wang, Y. (2020) Building a robust chromatin immunoprecipitation method with substantially improved efficiency. Plant Physiol. 183, 1026–1034. 32327547 10.1104/pp.20.00392 PMC7333696

[pbi70185-bib-0052] Zheng, Y. , Ge, J. , Bao, C. , Chang, W. , Liu, J. , Shao, J. and Zhou, D.X. (2020) Histone deacetylase HDA9 and WRKY53 transcription factor are mutual antagonists in regulation of plant stress response. Mol. Plant, 13, 598–611. 31891777 10.1016/j.molp.2019.12.011

[pbi70185-bib-0053] Zhu, G. , Jin, L. , Sun, W. , Wang, S. and Liu, N. (2022) Proteomics of post‐translational modifications in colorectal cancer: discovery of new biomarkers. Biochim. Biophys. Acta Rev. Cancer, 1877, 188735. 35577141 10.1016/j.bbcan.2022.188735

