# Supplementary figures and images for: The SCARECROW‐LIKE transcription factor from Populus davidiana × P. bolleana simultaneously improved drought tolerance and plant growth through acetylation‐dependent mechanisms
Source: Plant Biotechnol J. 2025 Jun 9;23(9):3650–66. doi: 10.1111/pbi.70185 (PMC12392933; doi:10.1111/pbi.70185)

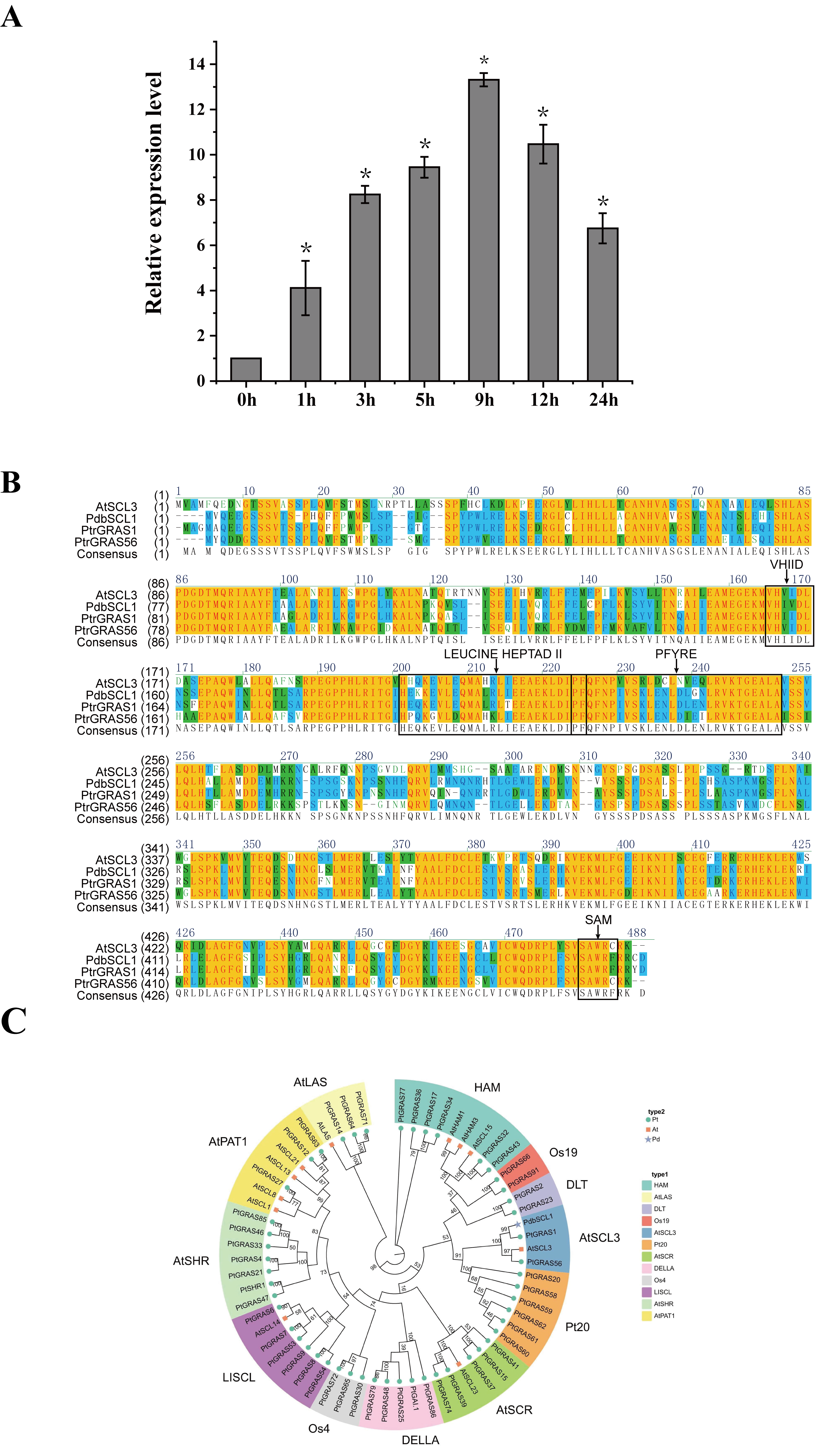

Supplement: Supplementary file 1 — Figure S1 Expression and bioinformatic analysis of PdbSCL1. Phylogenetic analysis of PdbSCL1 protein and GRAS transcription factor protein in Arabidopsis thaliana and Populus pilocarpa by MEGA6 neighbour‐joining method. (B) Multiple sequences analysis of GRAS proteins. A. thaliana (ATSCL3), P. trichocarpa (PtrGRAS1, PtrGRAS56). (C)The expression profiles of PdbSCL1 in response to PEG6000 treatment using RT‐qPCR. Plants were treated with 20%(W/V)PEG6000 for 0 (control), 1 3, 5, 9, 12, and 24 h. The expression of PdbSCL1 at 0 h was set as 1 to calculate the relative expression of PdbSCL1 at different stress time points. The error bar represents the standard deviation (SD) of three biological replicates. Asterisks indicate statistically signiffcant differences between 0 h and other times (*P < 0.05). [file PBI-23-3650-s002.tif]

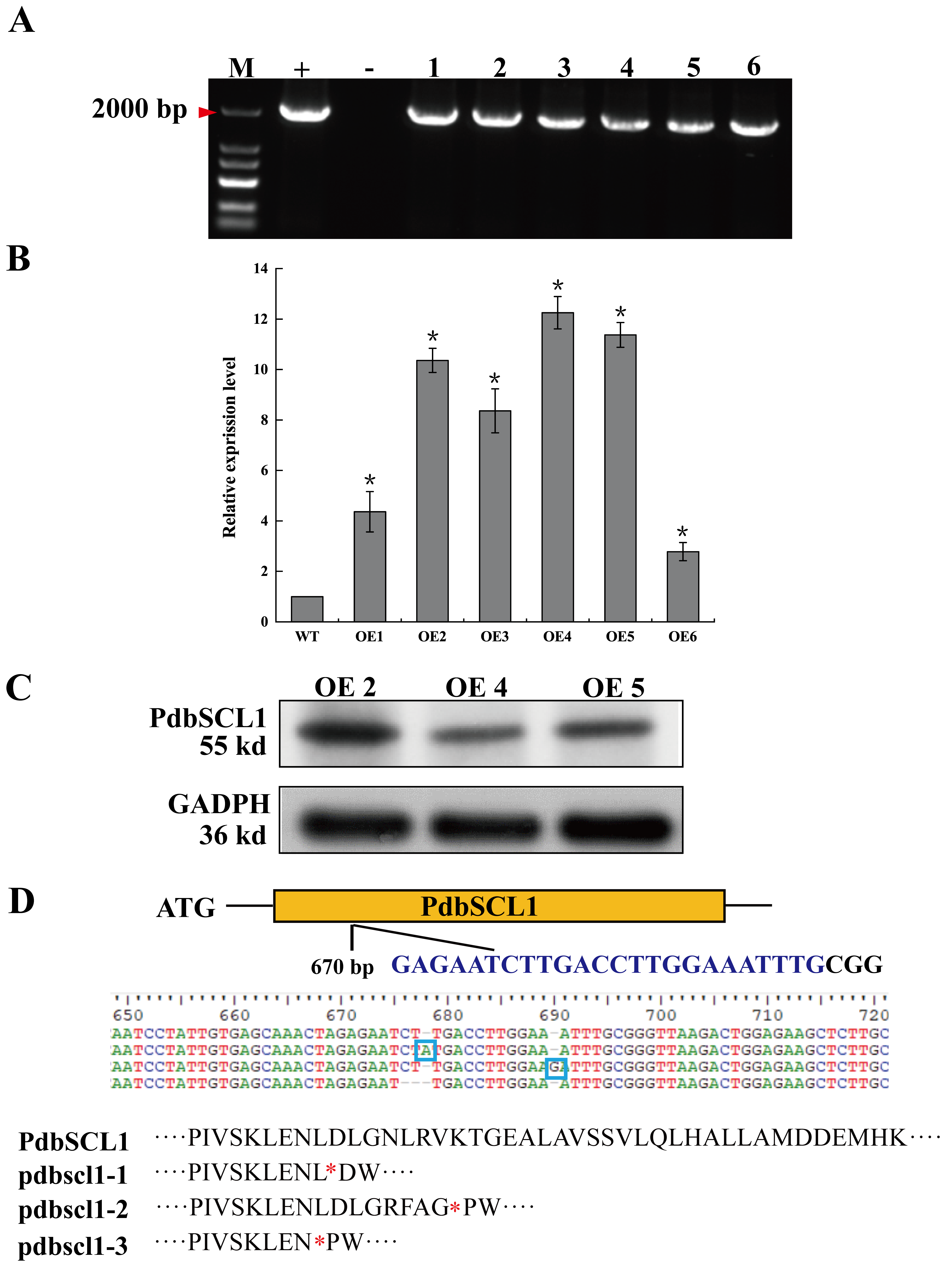

Supplement: Supplementary file 2 — Figure S2 Generation Populus davidiana × P. bolleana of lines with knockout, and overexpression of PdbSCL1. (A) PCR was used to detect whether PdbSCL1 was inserted into the genome. (B) RT‐qPCR analyses the expression of PdbSCL1 in different overexpression lines (OE). WT was used as a control and set to 1. The error bar represents the standard deviation (SD) of three biological replicates. Asterisks indicate a significant difference at P < 0.05. (C) Western blot was used to detect whether the gene is translated and expresses the correct protein. (D) Analysis of the mutation of PdbSCL1 (scl) induced by CRISPR using Sanger DNA sequencing. The target sequence and insertion nucleotides had been indicated. [file PBI-23-3650-s003.tif]

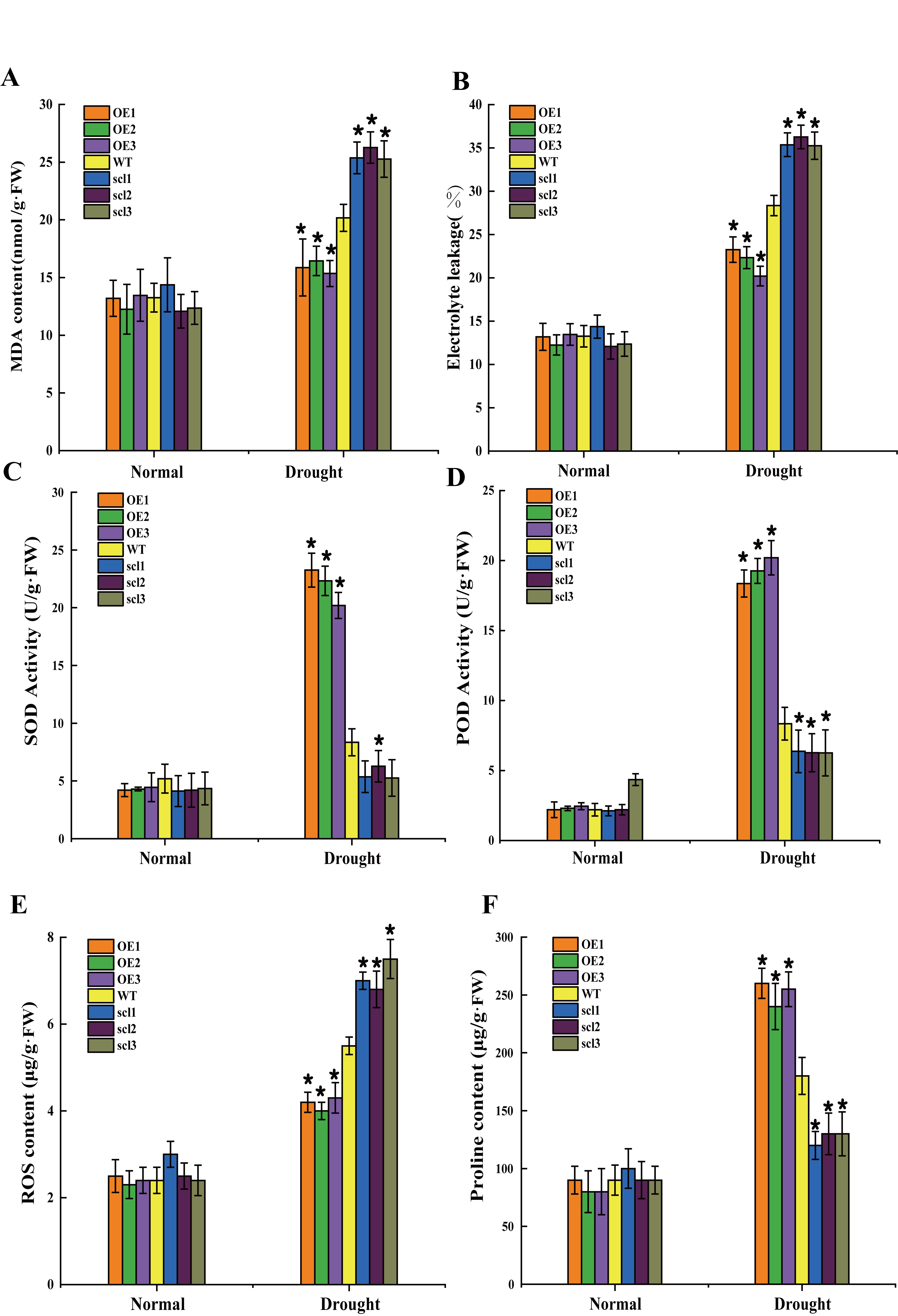

Supplement: Supplementary file 3 — Figure S3 Determination of physiological parameters involved in drought stress tolerance. (A) Measurement of MDA content. (B) Electrolyte leakage rates. (C) SOD activity. (D) POD activity. (E) ROS content. (F) Proline content. Data are presented as means ± SD from three independent experiments. Asterisks (*) indicate significant differences (P < 0.05) compared with the control under normal or drought conditions. OE, overexpressing PdbSCL1 Shanxin poplar lines; scl, Shanxin poplar with PdbSCL1 knockout induced by CRISPR/Cas; WT, wild‐type plants. ‘Normal’ indicates plants grown under standard conditions; ‘drought’ indicates plants treated with no water for 10 days. [file PBI-23-3650-s008.tif]

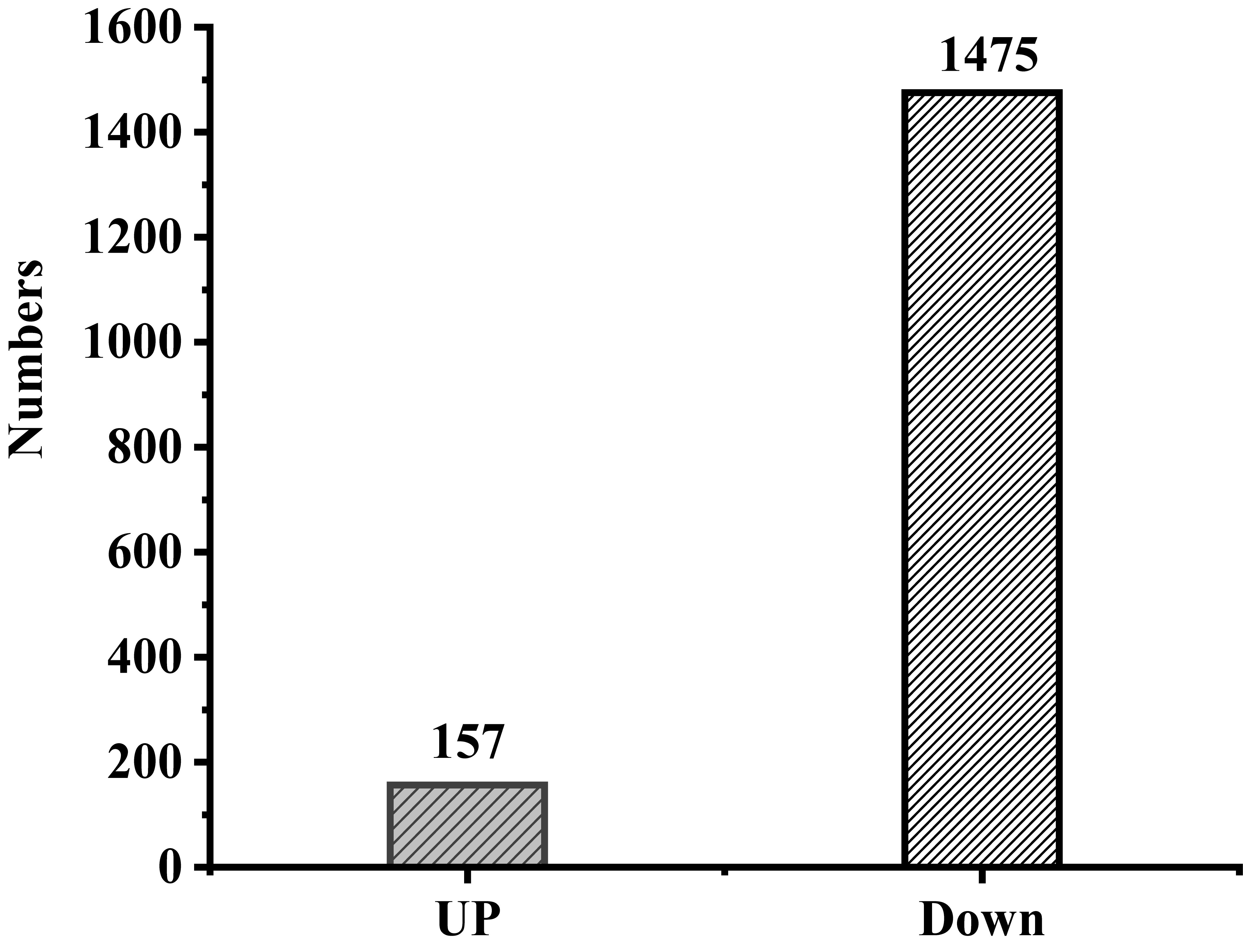

Supplement: Supplementary file 4 — Figure S4 Differentially expressed genes of PdbSCL1 followed by RNA‐seq analysis. The column diagram illustrates the number of differentially expressed genes (DEGs) between the PdbSCL1‐OE lines and the wild‐type (WT) following exposure to a 20% PEG6000 solution for 9 h. [file PBI-23-3650-s007.tif]

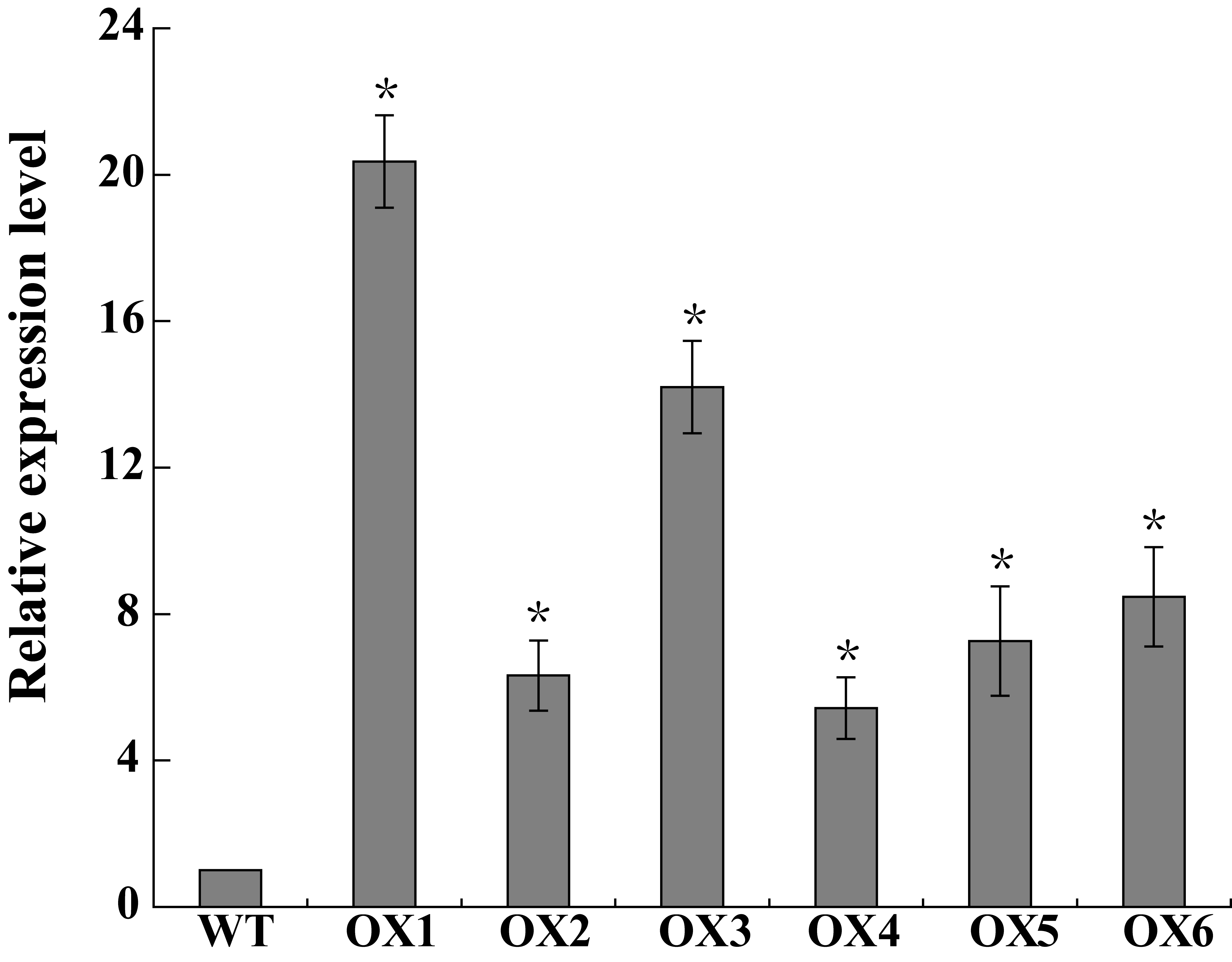

Supplement: Supplementary file 5 — Figure S5 RT‐qPCR analyses the expression of PdbSCL1K106/444R in different overexpression lines (OX). WT was used as a control and set to 1. The error bar represents the standard deviation (SD) of three biological replicates. Asterisks indicate a significant difference at P < 0.05. [file PBI-23-3650-s005.tif]

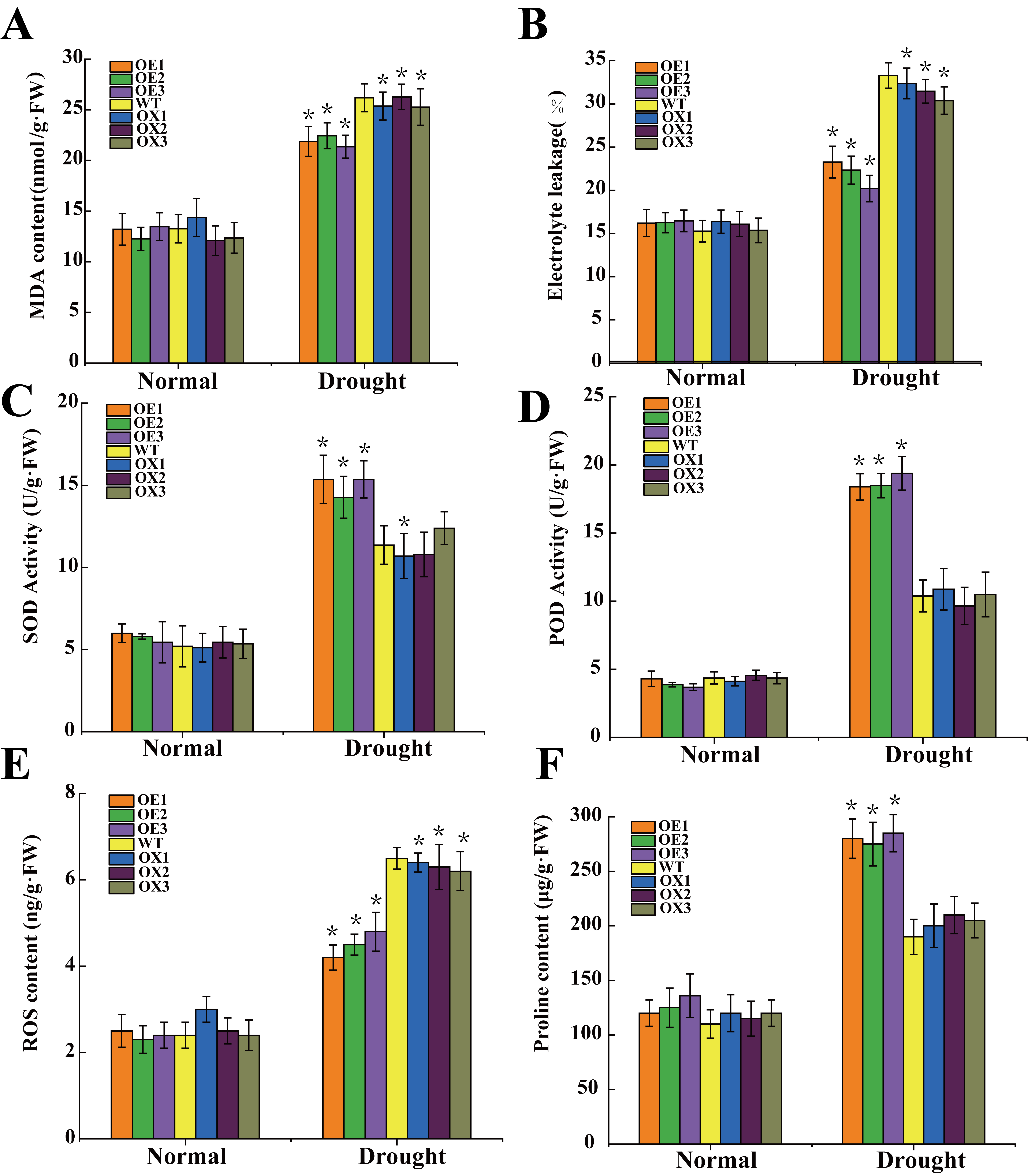

Supplement: Supplementary file 6 — Figure S6 Comparison of physiological parameters related to drought stress tolerance between PdbSCL1 and PdbSCL1K106/444R with mutated acetylation sites. (A) Measurement of MDA content. (B) Analysis of electrolyte leakage rates. (C) Assessment of SOD activity. (D) Measurement of POD activity. (E) Determination of ROS content. (F) Measurement of proline content. Data are presented as means ± SD from three independent experiments. Asterisks (*) indicate significant differences (t‐test, P < 0.05) compared with the control under normal or drought conditions. OE, Shanxin poplar lines overexpressing PdbSCL1; K106/444R1‐3, Shanxin poplar overexpressing PdbSCL1K106/444R; WT, wild‐type plants. ‘Normal’ indicates plants were grown under normal conditions; ‘drought’ refers to plants that were treated without water for a duration of 10 days. [file PBI-23-3650-s004.tif]
